# Evolution of age-related mutation-driven clonal haematopoiesis over 20 years is associated with metabolic dysfunction in obesity

**DOI:** 10.1016/j.ebiom.2023.104621

**Published:** 2023-05-18

**Authors:** Johanna C. Andersson-Assarsson, Rosanne C. van Deuren, Felipe M. Kristensson, Marloes Steehouwer, Kajsa Sjöholm, Per-Arne Svensson, Marc Pieterse, Christian Gilissen, Magdalena Taube, Peter Jacobson, Rosie Perkins, Han G. Brunner, Mihai G. Netea, Markku Peltonen, Björn Carlsson, Alexander Hoischen, Lena M.S. Carlsson

**Affiliations:** a Department of Molecular and Clinical Medicine, Institute of Medicine, The Sahlgrenska Academy at University of Gothenburg, Gothenburg, SE-405 30, Sweden; b Department of Human Genetics, Radboud University Medical Center, Nijmegen, 6525 GA, the Netherlands; c Department of Internal Medicine, Radboud University Medical Center, Nijmegen, 6525 GA, the Netherlands; d Radboud Institute for Molecular Life Sciences, Radboud University Medical Center, Nijmegen, 6525 GA, the Netherlands; e Radboud Expertise Center for Immunodeficiency and Autoinflammation and Radboud Center for Infectious Disease (RCI), Radboud University Medical Center, Nijmegen, 6525 GA, the Netherlands; f Region Västra Götaland, Sahlgrenska University Hospital, Gothenburg, SE-413 45, Sweden; g Institute of Health and Care Sciences, The Sahlgrenska Academy at University of Gothenburg, Gothenburg, SE-405 30, Sweden; h Department of Clinical Genetics, Maastricht University Medical Center, Maastricht, 6200 MD, the Netherlands; i Donders Institute for Brain, Cognition and Behaviour, Radboud University Medical Center, Nijmegen, 6525 GA, the Netherlands; j GROW School of Oncology and Developmental Biology, and MHeNs School of Mental Health and Neuroscience, Maastricht University, Maastricht, 6500 MD, the Netherlands; k Department of Immunology and Metabolism, Life and Medical Sciences Institute (LIMES), University of Bonn, Bonn, 53115, Germany; l Department of Neurobiology, Care Sciences and Society, Karolinska Institutet, Stockholm, SE-141 83, Sweden; m Public Health Promotion Unit, National Institute for Health and Welfare, Helsinki, 00271, Finland; n Translational Science and Experimental Medicine, Research and Early Development, Cardiovascular, Renal and Metabolism (CVRM), BioPharmaceuticals R&D, AstraZeneca, Gothenburg, SE-431 83, Sweden

**Keywords:** Clonal haematopoiesis, Clone size, Obesity, Bariatric surgery, HDL-Cholesterol, Insulin resistance

## Abstract

**Background:**

Haematopoietic clones caused by somatic mutations with ≥2% variant allele frequency (VAF) increase with age and are linked to risk of haematological malignancies and cardiovascular disease. Recent observations suggest that smaller clones (VAF<2%) are also associated with adverse outcomes. Our aims were to determine the prevalence of clonal haematopoiesis driven by clones of variable sizes in individuals with obesity treated by usual care or bariatric surgery (a treatment that improves metabolic status), and to examine the expansion of clones in relation to age and metabolic dysregulation over up to 20 years.

**Methods:**

Clonal haematopoiesis-driver mutations (CHDMs) were identified in blood samples from participants of the Swedish Obese Subjects intervention study. Using an ultrasensitive assay, we analysed single-timepoint samples from 1050 individuals treated by usual care and 841 individuals who had undergone bariatric surgery, and multiple-timepoint samples taken over 20 years from a subset (n = 40) of the individuals treated by usual care.

**Findings:**

In this explorative study, prevalence of CHDMs was similar in the single-timepoint usual care and bariatric surgery groups (20.6% and 22.5%, respectively, P = 0.330), with VAF ranging from 0.01% to 31.15%. Clone sizes increased with age in individuals with obesity, but not in those who underwent bariatric surgery. In the multiple-timepoint analysis, VAF increased by on average 7% (range −4% to 24%) per year and rate of clone growth was negatively associated with HDL-cholesterol (R = −0.68, 1.74 E^−04^).

**Interpretation:**

Low HDL-C was associated with growth of haematopoietic clones in individuals with obesity treated by usual care.

**Funding:**

The 10.13039/501100004359Swedish Research Council, The Swedish state under an agreement between the Swedish government and the county councils, the ALF (Avtal om Läkarutbildning och Forskning) agreement, The 10.13039/501100003793Swedish Heart-Lung Foundation, The 10.13039/501100009708Novo Nordisk Foundation, The 10.13039/501100000781European Research Council, The 10.13039/501100003246Netherlands Organisation for Scientific Research.

**Research in context**

**Evidence before this study**

Haematopoietic clones caused by somatic mutations with increase with age and are linked to risk of haematological malignancies and cardiovascular disease. Prevalence of clonal haematopoiesis is higher in individuals with obesity. Obesity is associated with metabolic dysregulation, which can be reduced by bariatric surgery.

**Added value of this study**

We examined clone size in individuals with obesity treated by usual care or bariatric surgery and evaluated clone growth over time.

We showed that clone size increases with age in individuals with obesity given usual care but not after bariatric surgery. Furthermore, clone growth was associated with low HDL-C in multiple-timepoint samples over 20 years.

**Implications of all the available evidence**

These results suggest that treatments improving metabolism may reduce age-related clone growth and thereby cardiovascular risk.

## Introduction

Clonal haematopoiesis-driver mutations (CHDMs) are somatic mutations that occur in haematopoietic stem cells (HSC) in the bone marrow and lead to clones detectable in peripheral blood cells.^1^^,^^2^ CHDMs have been described in several genes, with *DNMT3A, TET2, ASXL1,* and *JAK2* amongst the most frequently mutated.^1^^,^^2^ Genomic instability, including the accumulation of somatic mutations, is a hallmark of aging^3^ and the prevalence of CHDMs increases with age.^4^ Some studies suggest that clonal haematopoiesis may also be influenced by environmental stressors such as inflammation,^1^ and that cellular stressors may promote the expansion of mutant clones.^5^

Individuals with clonal haematopoiesis have increased risk of haematological cancers, but many CHDM carriers present without haematological diseases.^1^^,^^2^ When the variant allele frequency (VAF) of CHDMs in peripheral blood is ≥2% in the absence of haematological malignancy, the state is called clonal haematopoiesis of indeterminate potential (CHIP).^6^ More recently, the presence of clonal haematopoiesis has been associated with increased all-cause mortality and risk of several non-haematological diseases, including myocardial infarction, heart failure and type 2 diabetes^2^^,^^4^^,^^7^^,^^8^ and mechanistic studies in mice suggest that the relationship is causal.^8^, ^9^, ^10^ In individuals with clonal haematopoiesis, clone size appears to correlate to risk of disease^8^ with higher risk of both cardiovascular events and haematological malignancies in those with clones with VAF >10% compared to those with smaller clones.^4^ However, a recent study identified a lower VAF threshold that is associated with worse outcome in patients with heart failure.^11^

The risk of cardiometabolic diseases increases with age,^12^ an association that may be strengthened by obesity. Obesity and aging share many molecular and cellular changes and studies have shown that obesity reduces life expectancy by 5–20 years.^13^, ^14^, ^15^, ^16^ Recent work suggests that CHDM prevalence in otherwise healthy older women is higher in those with obesity compared to those with normal body weight,^17^ supporting the idea that obesity accelerates the age-related increase in clonal haematopoiesis. The mechanisms that link increased prevalence of CHDMs to obesity are unknown, but inflammation and metabolic abnormalities (e.g., dyslipidaemia and glucose dysregulation) that are associated with obesity could play a role. Furthermore, the drivers of clonal outgrowth over time are largely unknown, and novel, potentially environmental, triggers may be involved. Although most clones appear to be stable over time in healthy individuals,^18^ growth of individual clones has not been investigated in individuals with obesity.

It is largely unknown how clonal haematopoiesis is affected by factors other than age and if the somatic mutations can be influenced by interventions. Here we used an ultrasensitive assay to analyse clonal haematopoiesis in blood samples from well-characterized individuals with obesity treated by usual care or bariatric surgery in the Swedish Obese Subjects (SOS) study. The aims of this explorative study were to analyse haematopoietic clones of variable sizes, and to examine the expansion of clones over time in relation to age and metabolic dysregulation in individuals with obesity.

## Methods

### Subjects and samples

In this study, we used blood samples and data on clinical characteristics from the Swedish Obese Subjects (SOS) study. The SOS study was designed to compare long-term effects of usual care and bariatric surgery in patients with obesity, recruited between 1987 and 2001, as previously described.^13^ Inclusion criteria were age between 37 and 60 years and body-mass index (BMI) of ≥34 for men and ≥38 for women. Exclusion criteria were identical in the usual care and surgery groups and designed to exclude patients not suitable for surgery. Individuals in the usual care group were given conventional non-surgical obesity care, and individuals in the surgery group underwent bariatric surgery, with follow-up visits at 480 primary health care centres (usual care group) or at 25 surgery clinics (surgery group) at baseline (0), 0.5, 1, 2, 3, 4, 6, 8, 10, 15, and 20 years in both groups.

In the present study, clonal haematopoiesis was studied at a single timepoint during follow-up from 1050 individuals treated by conventional non-surgical obesity care (single-timepoint usual care group) and 841 individuals who had undergone bariatric surgery (single-timepoint bariatric surgery group) at least one year prior to blood sampling. DNA was extracted using the AGOWA mag kit (LGC Group, Teddington, Middlesex, UK). In addition, DNA was extracted from blood samples obtained from up to 5 timepoints over 20 years in 40 individuals (multiple-timepoint usual care group) using the DNeasy 96 Blood & Tissue kit (Qiagen, Hilden, Germany). These individuals were a subgroup of the single-timepoint usual care group in whom CHDMs had been detected and where repeated blood samples and information on metabolic parameters were available. In the resulting multiple-timepoint usual care group, 180 blood samples taken at baseline and at the 2-, 10-, 15-, and/or 20-year examinations (n = 40, 38, 40, 38, and 24, respectively) were available for DNA extraction.

### CHDM detection

CHDMs were analysed by an ultrasensitive assay using single-molecule molecular inversion probe (smMIP) sequencing, as previously described^19^ (Supplemental Figure S1). Briefly, smMIP sequencing of the entire *DNMT3A* gene as well as previously established CH-related hotspots in 23 additional genes was performed with two PCR and sequencing replicates for each DNA sample followed by two independent data-processing strategies and a targeted quality control to ensure high-quality. The average coverage for each of two technical replicates per sample was 2840× and 3891× for the single- and multiple-timepoint groups, respectively (see Supplemental Methods and Supplemental Table S1 for further details).

### CHDM definitions and classifications

In the single-timepoint groups, CHDMs were classified as small (VAF <2%) and large (≥2%) clones, as 2% is the cut-off commonly used to define CHIP.^6^ In the multiple-timepoint usual care group, CHDMs were classified based on their evolvement during follow-up. CHDMs present in at least three timepoints in an individual were classified as traceable trajectories, whereas CHDMs only observed at one or two timepoints were classified as events. Traceable trajectories were then further subclassified based on clone dynamics: 1) growing trajectories—where the absolute VAF of CHDMs at the final timepoint was at least 0.5% higher than at the first timepoint, 2) shrinking trajectories—where the VAF of CHDMs at the final timepoint was at least 0.5% lower than at the first timepoint, and 3) static trajectories—where the VAF of CHDMs at the final and first timepoints differed by less than 0.5%. As our trajectory definition requires CHDMs to be present at three or more timepoints, we annotated events with a VAF ≥2% at the final timepoint as ‘late-appearing clones’, as the abrupt appearance of a CHDM with such a high VAF likely suggests a fast-growing clone and therefore has the potential to be a growing trajectory.

### Statistical analyses

In the single-timepoint usual care and bariatric surgery groups, age differences were assessed using Wilcoxon-rank sum tests. Logistic regression models were used to determine the impact of age on CHDM prevalence, and a linear regression model was used for the effect of age on CHDM size (with log transformed VAF), for individuals with multiple clones, the clone with the largest VAF was used in this analysis.

The multiple-timepoint usual care group enabled us to determine the effect of age on clone growth, as dependent measurements allowed for tracing of a single clone over time. To this end, we first selected a single CHDM trajectory per individual. We hypothesized that trajectories with higher VAFs are more important than trajectories with lower VAFs, since literature indicates that clone size correlates to the risk of disease.^8^ The trajectory with the highest VAF at any timepoint was identified in each individual as the dominant trajectory (Supplemental Figure S2). Individuals in whom the dominant trajectory was shrinking were excluded from the subsequent analyses because shrinkage has been suggested to be caused by negative selection due to other (undetected) driver mutations.^20^ Age was used as a measure of time in mixed linear model analyses with random intercept and random slope to determine the effect of age on growth (VAF) at each timepoint (for details see supplement). Each individual has repeated measures on both VAF and age. The parameter estimate for age from the mixed model is therefore an estimate of the rate of growth; how the VAF changes over time. The model was built with clone size (VAF) as the dependent variable and age as explanatory variable. The random coefficient mixed model allows each individual to have a unique trajectory (how clone size changes with time/aging), and this can be estimated from the model. The estimated regression coefficient is then the rate of growth, unique for each individual. To assess potential factors that could underlie differences in effect size of age on rate of growth, we evaluated the role of clinical characteristics including insulin resistance (HOMA-IR)^21^ and HDL-C,^22^^,^^23^ variables related to leukocytosis and cardiovascular disease and, based on the outcome of this assessment, triglycerides^24^ and non-HDL cholesterol^25^^,^^26^ were also assessed. Finally, the resulting rates of growth (estimated regression coefficients) were correlated to clinical parameters averaged over the first three follow-up timepoints, by means of Spearman correlation coefficients, and subsequently the mixed linear model was expanded with significant averaged clinical parameters.

All statistical analyses were performed in R version 3.6.1 (R Core Team, URL https://www.R-project.org/), P-values <0.05 were considered statistically significant unless otherwise specified. For the correlations of clone growth with metabolic parameters, we performed Bonferroni-corrections for multiple-testing.

### Ethics

Seven regional ethics review boards (Gothenburg, Umeå, Uppsala, Karolinska Institute (Stockholm), Örebro, Linköping and Lund) approved the study protocol and written or oral informed content was obtained. Ethical approval reference numbers: 604-01 and T508-17. All clinical investigations were conducted according to the principles expressed in the Declaration of Helsinki.

### Role of funder

The funders had no role in the design and conduct of the study; collection, management, analysis, and interpretation of the data; preparation, review, or approval of the manuscript; and decision to submit the manuscript for publication.

## Results

### Subjects and samples

High-quality sequencing data was obtained from single-timepoint blood samples from the usual care (n = 1050) and bariatric surgery (n = 841) groups and from 180 multiple-timepoint blood samples from a subset of the usual care group (n = 40).

**Fig. 1:**
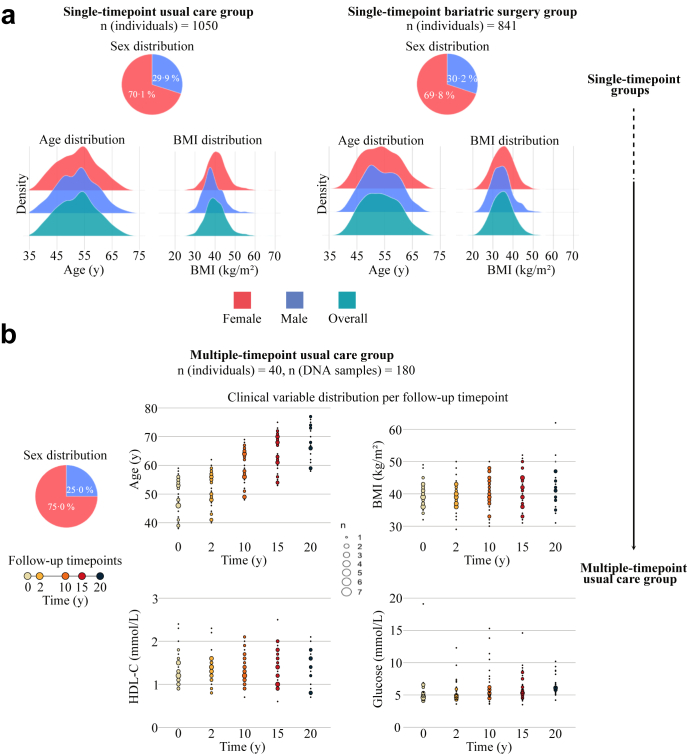
**Characteristics of the single- and multiple-timepoint groups. (a)** Sex, age, and BMI distribution in the single-timepoint groups (1050 + 841 individuals). **(b)** Sex distribution and distribution of clinical variables at the indicated follow-up timepoints in the multiple-timepoint usual care group (40 individuals).

Clinical characteristics of the single- and multiple timepoint groups at the time of DNA sampling are shown in Table 1. The age of individuals in the single-timepoint usual care group ranged from 37.3 to 70.2 years (mean 52.4 years) and women were overrepresented (70.1% vs 29.9% men, Fig. 1a). The age and sex distribution of the individuals in the single-timepoint bariatric surgery group was similar (38.9–69.8 years, mean 53.6 years; 69.8% women vs 30.2% men, Fig. 1a). The individuals in the single-timepoint bariatric surgery group had lower BMI and had less severe metabolic disturbances compared to the individuals in the single-timepoint usual care group, including lower insulin concentrations, lower homeostatic model assessment for insulin resistance (HOMA-IR) and higher HDL-C concentrations (Table 1).

**Table 1:** Characteristics at time of DNA in the single-timepoint usual care and bariatric surgery groups.

	Single-timepoint usual care	Single-timepoint bariatric surgery	P^a^
CHDM carriers, n (%)	216 (20.6)	189 (22.5)	0.338
Number of CHDMs, n (% of CHDM carriers)			0.202
1	172 (79.6)	165 (87.3)	
2	34 (15.7)	18 (9.5)	
3	7 (3.2)	5 (2.6)	
4	3 (1.4)	1 (0.5)	
CHIP, n (% of CHDMs)	71 (32.9)	66 (34.9)	0.663
Male sex, n (%)	59 (27.3)	59 (31.2)	0.452
Age, yrs. (mean (SD))	55.0 (6.98)	55.2 (6.66)	0.710
BMI, kg/m^2^ (mean (SD))	40.4 (5.26)	34.4 (5.28)	<0.001
Waist to hip-ratio (mean (SD))	0.98 (0.08)	0.95 (0.08)	0.001
Plasma glucose, mmol/L (mean (SD))	5.94 (2.38)	5.27 (1.71)	0.002
Type 2 diabetes^b^, n (%)	49 (22.8)	28 (14.8)	0.056
Insulin, mU/L (mean (SD))	17.3 (12.4)	11.9 (8.21)	<0.001
HOMA-index, mU/L∗mmol/L (mean (SD))	4.73 (4.13)	3.08 (3.51)	<0.001
Total cholesterol, mmol/L (mean (SD))	5.48 (1.05)	5.55 (1.05)	0.513
HDL-cholesterol, mmol/L (mean (SD))	1.38 (0.33)	1.61 (0.45)	<0.001
LDL-cholesterol^c^, mmol/L (mean (SD))	3.21 (0.86)	3.25 (0.98)	0.703
Triglycerides, mmol/L (mean (SD))	1.95 (1.37)	1.53 (0.78)	<0.001
High-sensitive CRP, mg/L (mean (SD))	7.89 (8.18)	4.52 (7.12)	<0.001
Systolic blood pressure, mmHg (mean (SD))	142 (18.7)	140 (18.0)	0.427
Diastolic blood pressure, mmHg (mean (SD))	82.9 (9.76)	85.3 (10.5)	0.019
Hypertension^d^, n (%)	150 (73.2)	134 (70.9)	0.697
Daily smoker, n (%)	54 (25.0)	49 (25.9)	0.921

a T-test for continuous variables, Chi^2^-test for categorical variables.

b Based on blood glucose and/or use of anti-diabetes medication.

c Based on Friedewalds equation: LDL-cholesterol = Total cholesterol−(Triglycerides/2.2)−HDL-cholesterol.

d Based on systolic blood pressure ≥140 or diastolic blood pressure ≥90 or self-reported use of blood pressure lowering medication.

For the 20-year multiple-timepoint usual care group with data from 40 individuals, DNA was available for analysis from five timepoints (baseline/0-, 2-, 10-, 15-, 20 years) for more than half of the individuals (n = 21), from four timepoints for 18 individuals, and from three timepoints for one individual. Baseline characteristics are shown in Supplemental Table S2. Women were again overrepresented (75.0% vs 25.0% men) (Fig. 1b). BMI, serum HDL-C, and plasma glucose levels remained relatively stable over 20 years (Fig. 1b).

### CHDM prevalence in the single-timepoint groups

In the single-timepoint usual care group, 216 of 1050 (20.6%) individuals carried one or more CHDMs, representing 135 unique mutations (Supplemental Table S3a). A single CHDM was detected in 172 individuals, and 44 individuals carried more than one CHDM. In the single-timepoint bariatric surgery group, 189 of 841 (22.5%) carried one or more CHDMs representing 124 unique mutations (Supplemental Table S3b). A single mutation was detected in 165 individuals, while 24 individuals carried more than one CHDM. In both single-timepoint groups, the majority of mutations identified have been previously described or are novel loss-of-function (LoF) mutations in genes with previously described LoF mutations (*DNMT3A, ASXL1, TET2*) (82.8% and 80.8% of the mutations in the usual care and bariatric surgery groups, respectively, Supplemental Table S4). Prevalence of CHDMs increased with age, and CHDM carriers were significantly older than non-carriers in both groups (54.6 vs 50.9 years, P = 2.43 E^−06^ and 54.7 vs 52.6 years, P = 0.57 E^−04^, for carriers vs non-carriers in the usual care and bariatric surgery groups, respectively).

### CHDM clone size in the single-timepoint groups

**Fig. 2:**
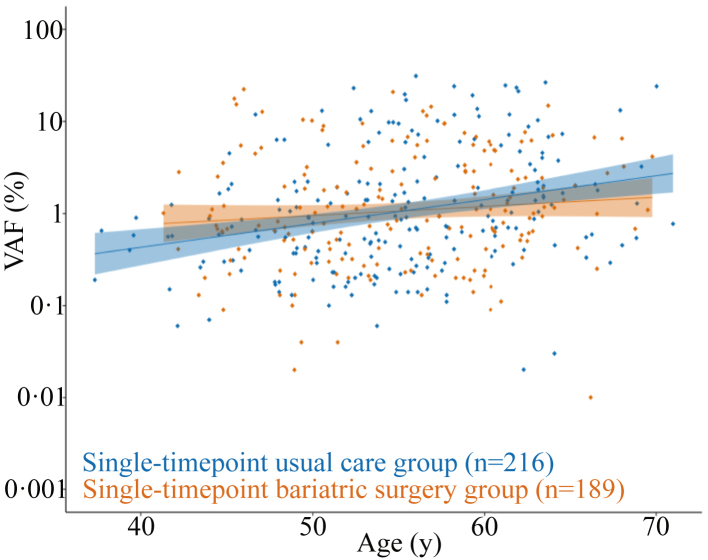
**CHDM size in relation to age in the single-timepoint usual care and bariatric surgery groups.** Comparison of the correlation between age and clone size (VAF) in individuals with obesity given usual care (shown in blue) and individuals with obesity that underwent bariatric surgery at least one year prior to DNA sampling (shown in orange).

Average clone size was similar in the two single-timepoint groups (mean VAF 2.72% [0.01–30.15] vs 2.44% [0.01–22.39], P = 0.474, in the usual care and bariatric surgery groups, respectively). In the single-timepoint usual care group, age was significantly associated with clone size (log-transformed VAF) with an effect estimate of 0.059 (P = 2.58 E^−05^), meaning that the average clone size in this group is 6% larger for each year of aging. However, in the single-timepoint bariatric surgery group, no significant association between age and clone size was detected (P = 0.184) (Fig. 2).

### Evolution of CHDMs over up to 20 years

A multiple-timepoint usual care group was created by selecting 40 individuals from the single-timepoint usual care group in whom we had identified at least one CHDM and for whom DNA was available at three or more timepoints over 20 years. Among these 40 individuals, we identified 115 CHDMs that were present at one or more timepoints and represented 53 unique mutations (Supplemental Table S5). Similar to the single-timepoint results, the majority of the unique mutations (73.6%) have been reported in the literature or represent LoF mutations (Supplemental Table S4).

Of the 115 identified CHDMs, 38 were only observed once or twice and defined as events, 16 of which disappeared at later timepoints, however, none of which reached CHIP level (VAF ≥2%) and the majority represented very small clones (9/16 with a VAF ≥0.1%). 77 were defined as traceable trajectories, with 22 individuals carrying more than one trajectory. Among the 77 traceable trajectories, we identified 30 growing, 42 static, and 5 shrinking trajectories (Supplemental Figure S3). None of the shrinking trajectories disappeared. Of the 42 static trajectories, the vast majority remained detectable while five did not fulfil the criteria for detection at the latest timepoint. In addition, three individuals had late-appearing clones—CHDMs that appeared at the last measured timepoint as a large clone (VAF ≥2%).

Among the 30 growing trajectories, 24 started as a small clone (VAF <2%) and nine of those had become large clones (VAF ≥2%, CHIP) at the last timepoint of detection. For the remaining six growing trajectories, the clone was already above the CHIP level at the first timepoint of detection. For the 42 static trajectories, all clones had VAF below CHIP level with the exception of one timepoint in one individual. Of the five shrinking CHDM trajectories, three started as large clones, two of which shrunk below CHIP level (Supplemental Figure S3).

### Clone growth over 20 years

**Fig. 3:**
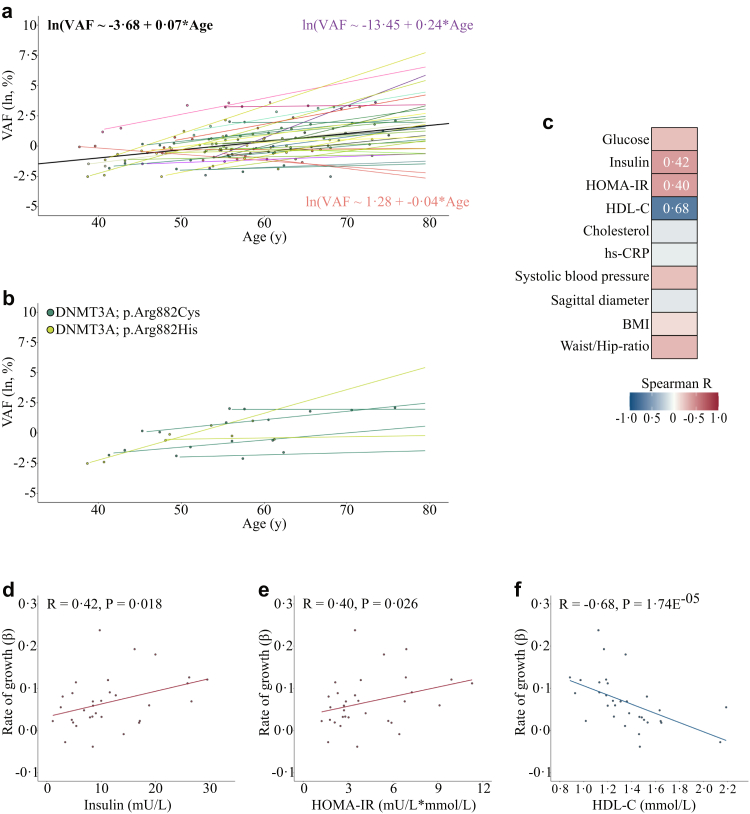
**Rate of CHDM growth in relation to age and metabolic dysfunction. (a)** VAF in relation to age for the 32 most important trajectories. The average mixed linear model (MLM) regression line and equation is shown in black. All individual MLM regression lines are shown in various colours, in purple the trajectory with maximum slope and accompanying equation, in orange the trajectory with minimum slope and accompanying equation. **(b)** VAFs and slopes for CHDMs in *DNMT3A* [p.(Arg822Cys)] in 4 individuals and *DNMT3A* [p.(Arg882His)] in 2 individuals. **(c)** Heatmap of Spearman R correlations between all individual trajectory effect estimates from our MLM to averaged (based on the three first follow-up timepoints) metabolic clinical parameters. **(d)** Positive Spearman R correlation between individual trajectory effect estimates and insulin. **(e)** Positive Spearman R correlation between individual trajectory effect estimates and HOMA-index. **(f)** Negative Spearman R correlation between individual trajectory effect estimates and HDL-C.

The multiple-timepoint usual care group enabled us to go beyond clone size and examine a potential association between age and actual clone growth. As some individuals carried multiple trajectories over the course of 20 years, we identified the dominant^27^ trajectory per individual by selecting trajectories with the largest VAF at any timepoint and excluding individuals with shrinking trajectories (n = 5) (Supplemental Figure S2). Because we expected variation in growth patterns, we used a mixed linear model including random intercept and random slope to determine the association between age and clonal growth. This model uses age as a measure of time and, since each individual has repeated measures of both VAF and age, the parameter estimate for age from the mixed model is an estimate of the rate of growth (how VAF changes over time). The average proportionate increase of VAF was 7% per year, ranging from −4% to 24% (Fig. 3a, three static clones had slightly lower VAF at the final timepoint compared to the first timepoint, explaining why the range includes values below zero), confirming the expected differences in rate of growth per trajectory. These differences in clone growth between individuals were also observed for identical mutations. For example, trajectories of *DNMT3A* [p.(Arg882Cys)], identified in four individuals, and *DNMT3A* [p.(Arg882His)], identified in two individuals, increased with different rates (Fig. 3b).

### Clone growth in relation to metabolic status

To examine if different rates of growth over time (as expressed by the effect of aging) associate with metabolic status, the individual trajectory effect estimates from the mixed linear model were correlated to cardiovascular risk factors averaged over the first three follow-up timepoints in univariate analyses (Fig. 3c). Rate of growth correlated positively with insulin (Spearman R = 0.42, P = 0.018) and insulin resistance measured by HOMA-IR (Spearman R = 0.40, P = 0.025), and negatively with HDL-C (Spearman R = −0.68, P = 0.74 E^−05^) (Fig. 3d–f), and after Bonferroni-correction for multiple-testing the negative correlation between rate of growth and HDL-C remained significant (P = 0.74 E^−04^). In addition, we performed a sensitivity analysis including the five individuals with shrinking clones and the significant correlation with HDL-C persisted (Spearman R = −0.43, P = 0.008). Given that low HDL is associated with elevated triglyceride-rich lipoproteins,^24^ and that non-HDL cholesterol represents a robust index of the total concentration of potentially atherogenic particles,^25^^,^^26^ we performed secondary explorative evaluation of triglycerides and non-HDL cholesterol. However, rate of growth did not significantly associate with triglyceride levels (Spearman correlation: R = 0.22; P = 0.22), nor with non-HDL cholesterol (Spearman correlation: R = −0.011; P = 0.954).

## Discussion

Clonal haematopoiesis is associated with increased risk of cardiovascular events, and both the presence of somatic mutations and clone size are important indicators of this risk.^8^ In this study, we examined clonal haematopoiesis in individuals with obesity and showed that clone growth is positively associated with metabolic dysfunction. In our single-timepoint groups, we first observed a significant association between clone size and age in individuals with obesity treated with usual care but not in those who had undergone bariatric surgery, a treatment that markedly improves metabolic status.^28^ Tracing of CHDMs over time in our multiple-timepoint usual care group subsequently revealed associations between clone growth and low HDL-C, supporting our hypothesis that dysfunctional metabolism affects clone growth.

Obesity is a complex phenotype, often associated with a wide range of metabolic abnormalities and diseases as well as accelerated aging and shortened lifespan. Accumulating evidence shows that there must be factors beyond traditional risk factors that contribute to the link between obesity and cardiovascular disease.^1^^,^^29^ It is therefore tempting to speculate that clonal haematopoiesis could contribute to increased cardiovascular disease and decreased life expectancy in people with obesity. We have previously shown that bariatric surgery reduces the incidence of cardiovascular disease^30^ and increases life expectancy^13^ in individuals with obesity. Our current study suggests that bariatric surgery could, at least partly, mediate these beneficial effects by improving metabolism and thereby mitigating the age-related increase in average clone size.

The importance of clonal haematopoiesis has mainly been studied for mutations above the CHIP level of 2% because of 1) methodological difficulties in detecting small clones and 2) the assumption that clones must reach a critical size to be clinically meaningful. CHIP mutations are associated with a wide range of diseases, including haematological malignancies and cardiovascular disease.^4^ The health consequences of smaller clones are largely unknown, but a recent study in patients with chronic ischemic heart failure indicated that CHDMs with VAF significantly lower than the CHIP level are associated with lower survival.^11^ The ultrasensitive assay used in our study allowed detection of small clones, and almost 70% of CHDMs in the single-timepoint groups were below the CHIP level definition. Irrespective of clone size, most CHDMs detected overlapped with those that have been described in the literature, however our targeted assay had a limited capability to identify novel mutations.

In the multiple-timepoint usual care group, nine out of 30 growing trajectories were initially small clones but grew over the sampling period to beyond the CHIP level. The longitudinal data thus showed that clones can be detected many years before they reach the CHIP level. This is in line with recent observations that clones responsible for clonal haematopoiesis in older individuals can be detected as small clones at earlier timepoints.^31^^,^^32^

A unique feature of our study was the collection of up to five samples taken over 20 years from 40 well-characterized individuals with obesity. Availability of these samples allowed measurement of clonal evolution over time and determination of actual clone growth in relation to clinical characteristics. The average annual growth rate of specific clones within individuals was 7%, which is within the broad range of clone growth rates (5–20% per year) reported for other cohorts of individuals with no history of haematological malignancy,^27^^,^^32^ but higher than that reported in individuals with atherosclerosis.^33^ However, there were large inter-individual differences in the rate of clone growth, even between individuals sharing the same CHDM, suggesting that factors other than the mutation itself influence clone growth. In agreement with this concept, a recent study used the term unknown-cause (growth) effect to describe the fraction of clone growth that is explained by factors other than the driver mutation and differs between individuals.^27^

A recent study showed that humans with atherosclerosis have increased HSC proliferation, which may in turn promote clonal haematopoiesis by increasing the risk of acquiring CHDMs and facilitating the expansion of mutant clones.^26^ Leukocytosis, an indicator of increased HSC proliferation, is associated with both insulin resistance^21^ and low levels of HDL-C.^22^^,^^23^ These studies are consistent with our longitudinal observations that increased rate of growth was associated with low HDL-C and possibly with insulin resistance.

It is possible that the observed association between metabolic dysfunction and clone growth is linked to increased HSC proliferation by directly influencing function and stability of CHIP-related proteins, including TET2.^34^^,^^35^ A vicious cycle has been proposed, whereby dysfunctional metabolism increases the risk of developing pro-inflammatory CHIP that, once formed, further increases the risk of worsening insulin resistance and atherosclerosis.^36^ Such a cycle is supported by our results together with earlier studies showing that loss of *Tet 2* function in HSCs leads to increased atherosclerosis^8^^,^^10^ and insulin resistance.^9^ Of relevance in terms of obesity, adipose tissue-derived interleukin (IL)-1β has been shown to stimulate proliferation of bone marrow progenitor cells.^37^ Inhibition of IL-1β reduces leukocytosis,^38^ providing clinical support that this pathway would be important to investigate in relation to clone growth in humans.

The increased risk associated with CHIP for several severe diseases and overall mortality raises the question of how this risk could be mitigated. In individuals with established CHIP, interventions may target the pro-inflammatory activity.^2^^,^^39^ Mice with experimental CHIP show increased pro-inflammatory drive of the NLRP3/IL-1β/IL-6 axis and atherosclerosis,^29^^,^^40^ and humans carrying a genetic deficiency in *IL6* appear to have reduced cardiovascular risk related to CHIP.^41^ Thus, intervention with IL-6 inhibitors could potentially reduce the risk of cardiovascular disease in patients with CHIP.

Our study is limited by the lack of individuals with normal body weight for comparison. Our conclusions are therefore related to the relationship between clonal expansion and metabolic disturbances in individuals with obesity, not the impact of obesity itself. Furthermore, we observed correlations, but our results do not allow us to deduct causality between clone growth and metabolic dysfunction. Strengths of this study are the ability to detect small clones, the possibility to examine the impact of an intervention that improves metabolism, and the longitudinal measurements, which allowed us to examine how CHDMs develop over up to 20 years and to examine the association with clinical characteristics. Due to the limited sample size of this explorative study, we are underpowered to analyse the impact of metabolic factors on shrinking clones and the association between clone expansion and hard cardiovascular endpoints. Larger longitudinal studies are needed to address these questions and to confirm our observation that low HDL-C, and possibly insulin resistance, are associated with clone growth.

In conclusion, in this study there was a significant age-related increase in clone size in individuals with obesity treated by usual care, but not in those with improved metabolism after bariatric surgery. Furthermore, low HDL-C was associated with enhanced clone growth. Together, these findings suggest that metabolic dysregulation affects clonal haematopoiesis. Further studies are needed to confirm this link and possible effects on clonal haematopoiesis by interventions that improve metabolic control.

## Contributors

B.C., A.H. and L.M.S.C. conceptualized the project, J.C.A.-A., R.C.vD., F.M.K., M.S., K.S., P-A.S., M.Pi., C.G., M.T., P.J., R.P., H.G.B., M.G.N., M.Pe., B.C., A.H., L.M.S.C. contributed intellectually to the study and the preparation of the manuscript. J.C.A.-A., R.C.vD., F.M.K., A.H. and L.M.S.C. wrote the first draft of the manuscript and all authors contributed to data interpretation, discussion and revised the manuscript. J.C.A.-A., K.S., P-A.S., M.T., P.J. and L.M.S.C. were responsible for collection of data and samples. R.C.vD., M.S. and A.H. generated and analysed sequencing data. J.C.A.-A., R.C.vD. and M.Pe. did the statistical analyses. J.C.A.-A. and R.C.vD. designed the figures. All authors approved the final manuscript. A.H. and L.M.S.C. are the guarantors of this work and, as such, had full access to all the data in the study and takes responsibility for the integrity of the data and the accuracy of the data analysis.

## Data sharing statement

All data reported in this study cannot be deposited in a public repository because data are subject to legal restrictions according to national legislation. Confidentiality regarding personal information in studies is regulated in the Public Access to Information and Secrecy Act (SFS 2009:400), OSL. A request to get access to public documents can be rejected or granted with reservations by the University of Gothenburg. If University of Gothenburg refuses to disclose the documents the applicant is entitled to get a written decision that can be appealed to the administrative court of appeal.

Code for processing and filtering smMIP-based sequencing data is explained in the Supplemental information of this manuscript. An optimized, automated version of this workflow is publicly available on GitHub: https://github.com/RosanneVanDeuren/CHMIP-RsCh-PIPELINE. The source code from the R-packages that were used in this study are freely available online (https://cran.r-project.org/).

## Declaration of interests

Dr F.M. Kristensson is supported by a grant from the Swedish State under an agreement between the Swedish government and the county councils, the ALF (Avtal om Läkarutbildning och Forskning) agreement, grant ALFGBG-970993 and has received a travel grant from the Gothenburg Society of Medicine. Ms. M. Steehouwer, Dr C Gilissen, Dr H.G. Brunner, and Dr A. Hoischen are supported by the Solve-RD project. The Solve-RD project has received funding from the 10.13039/501100007601European Union’s Horizon 2020 research and innovation programme under grant agreement No. 779257. Dr C. Gilissen is a member of the board of the European Society of Human Genetics (ESHG) and a member of the scientific programme committee of ESHG. Dr H.G. Brunner is a co-coordinator of the Solve-RD project. Dr M.G. Netea is a scientific founder of and owns stock in Trained Therapeuticx Discovery, has received an unrestricted grant from ViiV HealthCare, and a Spinoza Grant from The 10.13039/501100003246Netherlands Organisation for Scientific Research. Dr B. Carlsson is employed by and owns stock in AstraZeneca. Dr L.M.S. Carlsson has received consulting fees from Johnson & Johnson. All other authors declare they have no competing interests.
