# Supplementary material for: Evolution of age-related mutation-driven clonal haematopoiesis over 20 years is associated with metabolic dysfunction in obesity
Source: eBioMedicine. 2023 May 18;92:104621. doi: 10.1016/j.ebiom.2023.104621 (PMC10209127; doi:10.1016/j.ebiom.2023.104621)
Supplement: Supplemental Figures S1–S3 and Tables S1–S5 [file mmc1.pdf]

SUPPLEMENTAL INFORMATION

Table of contents:

Supplemental Methods

Swedish Obese Subjects (SOS) study

Inclusion and exclusion criteria

CHDM detection by single-molecule Molecular Inversion probe (smMIP) sequencing

Sequencing

Variant calling

Quality control single-timepoint groups

Quality control multiple-timepoint group

Statistical analyses

Mixed linear model to estimate rate of growth

Supplemental Tables

Table S1: Sequenced gene regions

Table S2 Baseline characteristics of the 40 individuals in the multiple-timepoint usual care group

Table S3a: CHDMs identified in the single-timepoint usual care group

Table S3b: CHDMs identified in the single-timepoint bariatric surgery group

Table S4a: CHDMs in literature – summary of studies used for comparisons shown in Tables S4b-d

Table S4b: Overlap of CHDMs in single-timepoint usual care group and CHDMs in literature (shown in Table 3a)

Table S4c: Overlap of CHDMs in single-timepoint bariatric surgery group and CHDMs in literature (shown in Table 3a)

Table S4d: Overlap of CHDMs in multiple-timepoint usual care group and CHDMs in literature (shown in Table 3a)

Table S5: CHDMs identified in the multiple-timepoint usual care group

Supplemental Figures

Figure S1. smMIP workflow to further boost sensitivity for low-level somatic mutation detection.

Figure S2. Trajectory selection flowchart.

Figure S3. Categorisation of CHDMs over 20 years.

Supplemental References

Supplemental Methods

Swedish Obese Subjects (SOS) study

Inclusion and exclusion criteria

Inclusion and exclusion criteria were identical for all individuals considered for participation in the SOS study. Exclusion criteria were selected to enrol patients who could undergo surgery and the same criteria were applied to the usual care group.
Among eligible individuals, individuals electing surgery constituted the surgery group and a contemporaneously matched usual care group was created by an automatic matching program.

Exclusion criteria were earlier operation for gastric or duodenal ulcer, earlier bariatric surgery, gastric ulcer during the past 6 months, ongoing malignancy, active malignancy during the past 5 years, myocardial infarction during the past 6 months, bulimic eating pattern, drug, or alcohol abuse, psychiatric or cooperative problems contraindicating bariatric surgery, other contraindicating conditions (such as continuous glucocorticoid or anti-inflammatory treatment).

CHDM detection by single-molecule Molecular Inversion probe (smMIP) sequencing

Sequencing

CHDMs were analysed by ultra-sensitive sequencing, as essentially previously described(1). We modified the existing assay by removing non-CH-related hotspot targets and designing additional double tiling smMIP probes(2) for the entire coding sequence of the most prevalently reported CH-driver gene *DNMT3A*. The final assay consisted of a total of 300 smMIP probes, with 54 nucleotide target sequence per probe, spanning a total of 7612 bases of target sequence (see Supplemental Table S2 for a list of genes, bases and target hotspots covered). smMIP captures were likewise performed with slight modifications: 200-300ng gDNA of each sample with a DNA to smMIP ratio of 1:2,400; all samples were captured twice, and each replicate was tagged with an independent barcode by PCR. Sequencing was performed in batches of up to 380 samples per run by 2x79 basepair PE reads on a high-output run on a NextSeq500 instrument (Illumina) (Supplemental Figure S1a).

Variant calling

For the purpose of providing true positive somatic variant calls, we applied two independent data processing strategies followed up by targeted quality control, specifically designed for this study (Supplemental Figure S1b).

Here we combine advantages of these two processing strategies:
1) Read count and quality check with an openly available software (bwa-mem mapping & mpileups), relying on standardized parameters, and benefitting from genome wide read data mapping that avoid any ambiguity for homologous sequences.
2) Consensus calling by sequencing of the UMI tag-groups (utilizing the commercial software JSI Sequence Pilot).

This approach combined the high accuracy of consensus calling (due to the error-correction capability) of CHDM candidates (strategy 1), with complementary information about coverage as well as base and read mapping quality (strategy 2), that cannot easily be retrieved from the proprietary algorithms of JSI Sequence Pilot.

Specifically, FASTQ files were: 1) aligned to the entire reference genome (Hg19) with BWA-MEM(3), and 2) imported into the commercially available NGS software package Sequence Pilot (JSI Medical Systems), using the optimized smMIP analysis module as described previously(4,5). The latter allows for a consensus calling per smMIP probe, enhancing individual variant quality by reducing random PCR or sequencing artefacts, using a majority vote of Unique Molecular Identifier (UMI) duplicates. This also enabled the same molecule to be read with forward and reverse sequencing reads, due to 2x79 basepair reads and a 54 nucleotide insert size of gDNA. Variant calling with Sequence Pilot was performed with the following settings: Minimum combined forward and reverse coverage was set to 10 reads, mutation calling required at least 5 consensus reads (forward and reverse reads considered separately) without a minimal % of variant reads, enabling some somatic calls down to 0·01% (depending on locus specific coverage); consensus calling was done with a minimum of 2 consensus UMI reads and by ignoring consensus read threshold of <30% as ‘likely artefacts'; and UMI-tags with “N" bases or low quality were ignored.

Quality control single-timepoint groups

The resulting variant calls were then subjected to the following stringent quality filtering steps (Supplemental Figure S1c): First, individuals with an average coverage below 500x based on the untargeted aligned BAM files were excluded (STEP1). Second, only variants called in both technical replicates were kept (STEP2). Third, the remaining duplicate variant calls were further filtered by excluding non-coding, synonymous, and likely germline (variant allele frequency (VAF) ≥40%) variant calls (STEP3). Fourth, variants called in >5% of the individuals that are considered likely run-specific artefacts (excluding most common known drivers) and common smMIP-run artefacts (based on previously processed smMIP-data) were excluded (STEP4). Fifth, remaining variant calls were flagged based on the following characteristics; a) *PTPN11* variants were excluded, due to mapping issues related to homology with various regions in the genome, b) variants with unspecified alternative allele by JSI (N-allele) were excluded, c) variants called in four or more samples with an alternative allele count below 16 when considering forward and reverse reads separately (based on JSI parameters) were excluded, and d) variants called in less than four samples with an alternative allele count below 24 were excluded, and e) based on visual inspection combined with previous validations(1) we flagged likely true positive and likely false positive variants in green and red respectively, excluding all variants with a red flag, overruling any of the previously described flags. Finally, the percentage of alternative alleles for the remaining variant calls was generated using SAMtools mpileup(6) on the untargeted aligned BAM files. Inconclusive mpileups, due to different indexing or complex variant calls, were checked manually, and were excluded if read-end or -start marker was present in the mpileup sequence. The resulting mpileup percentage provided the final VAF for our variant calls and was used in all subsequent analyses.

Quality control multiple-timepoint usual care group

To identify trajectories, i.e., the same clone in 3 or more timepoints in the same individual, we initially relied upon the same stringent variant calling that we used for the single-timepoint measures. Specifically, the data in the multiple-timepoint usual care group was subjected to the same quality filtering pipeline, with exception of the run-specific threshold in STEP3 as multiple timepoints of the same individuals constituted one run. The final output from STEP6 was used to trace CHDM calls per individual over all available timepoints, to allow most sensitive detection of the same CHDMs appearance at previous timepoints. However, as these supplemental mpileups were not initially identified as CHDMs by JSI Medical Systems Sequence Pilot, our two-fold CHDM detection approach is violated. We therefore added a level of stringency to the parsing of multiple-timepoint mpileups in terms of 1) a position-based coverage threshold of ≥500x, and 2) a minimum alternative allele count threshold of ≥3. For individuals in which we identified a CHDM that was not detected at other timepoints using the stringent calling strategy, we examined the mpileup data for all other timepoints. This allowed tracing of individual clones at additional timepoints, with the thresholds stated above.

Statistical analyses

Mixed linear model to estimate rate of growth

The growth rate was calculated in R with a mixed linear model using the lme-function and REML method from the nlme package.

R-script:

```
MLM_data = lme(log(VAF) ~ age, random=~age|sampleVariantKey, data=data, method=”REML”)
```

where sampleVariantKey identifies each individual.

Supplemental Tables

Table S1: Sequenced gene regions

Gene	CHR	BPstart	BPstop	Hotspot(s)
ASXL1	chr20	31021185	31021291	R404; R417
ASXL1	chr20	31022246	31022314	Y591
ASXL1	chr20	31022573	31022637	Y693
ASXL1	chr20	31022891	31022956	Q803
BRAF	chr7	140453103	140453168	V600
BRAF	chr7	140481373	140481439	G469
BRAF	chr7	140494133	140494201	R362
BRCC3	chrX	154305462	154305526	R81
CBL	chr11	119148879	119149040	C381; C396; C404
CBL	chr11	119149194	119149295	F418
DNMT3A	chr2	25457097	25457337	R882; F868
DNMT3A	chr2	25458534	25458737	W860
DNMT3A	chr2	25459766	25459898	
DNMT3A	chr2	25461963	25462122	P777
DNMT3A	chr2	25463116	25463351	R771; G762; Y735
DNMT3A	chr2	25463462	25463661	
DNMT3A	chr2	25464379	25464623	
DNMT3A	chr2	25466728	25466891	
DNMT3A	chr2	25466975	25467236	
DNMT3A	chr2	25467371	25467573	
DNMT3A	chr2	25468094	25468246	
DNMT3A	chr2	25468860	25468966	
DNMT3A	chr2	25468999	25469230	
DNMT3A	chr2	25469451	25469670	
DNMT3A	chr2	25469890	25470072	
DNMT3A	chr2	25470425	25470644	R326
DNMT3A	chr2	25470847	25471147	
DNMT3A	chr2	25497766	25497976	
DNMT3A	chr2	25498336	25498443	
DNMT3A	chr2	25505264	25505647	
DNMT3A	chr2	25522970	25523163	
DNMT3A	chr2	25536724	25536884	
FGFR2	chr10	123279627	123279726	P253
FGFR3	chr4	1803523	1803588	S249
FGFR3	chr4	1806082	1806145	G380
FGFR3	chr4	1807841	1807923	K650
GNAS	chr20	57484390	57484467	R844
GNB1	chr1	1747219	1747281	K57

HRAS	chr11	534241	534335	G12
IDH2	chr15	90631915	90631981	R140
JAK2	chr9	5073728	5073801	V617
KRAS	chr12	25398233	25398313	G12
MYD88	chr3	38182285	38182347	L265
MYD88	chr3	38182614	38182677	L273
NRAS	chr1	115256481	115256578	Q61
NRAS	chr1	115258695	115258772	G13
PIK3CA	chr3	178916710	178916775	R38
PTEN	chr10	89692869	89692955	R130
PTPN11	chr12	112888146	112888211	Y63
SF3B1	chr2	198266793	198266879	K700
SF3B1	chr2	198267310	198267375	K666
SRSF2	chr17	74732933	74733004	P95
STAT3	chr17	40474385	40474451	D661
TET2	chr4	106156676	106156762	L532; R550
TET2	chr4	106157359	106157422	Q764
TET2	chr4	106157586	106157655	H839
TET2	chr4	106157712	106157776	Q888
TP53	chr17	7577460	7577612	I255; G187; R248
TP53	chr17	7578157	7578226	Y220
TP53	chr17	7578374	7578461	R175
TP53	chr17	7579327	7579393	R110
U2AF1	chr21	44524436	44524500	S34

Table S2: Baseline characteristics in the multiple-timepoint usual care group

	**Multiple-timepoint usual care**
N	40
Male sex, n (%)	9 (22·5)
Age, yrs (mean (SD))	48·8 (6·2)
BMI, kg/m^2 (mean (SD))	39·0 (3·8)
Waist to hip-ratio (mean (SD))	0·98 (0·07)
Plasma glucose, mmol/L (mean (SD))	5·44 (2·35)
Type 2 diabetes[a], n (%)	2 (5·0)
Insulin, mU/L (mean (SD))	16·8 (9·4)
HOMA-index, mU/L*mmol/L (mean (SD))	4·11 (2·71)
Total cholesterol, mmol/L (mean (SD))	5·79 (1·23)
HDL-cholesterol, mmol/L (mean (SD))	1·34 (0·36)
LDL-cholesterol[b], mmol/L (mean (SD))	3·36 (1·02)
Triglycerides, mmol/L (mean (SD))	2·33 (2·96)
High-sensitive CRP, mg/L (mean (SD))	6·51 (4·23)
Systolic blood pressure, mmHg (mean (SD))	137·1 (15·1)
Diastolic blood pressure, mmHg (mean (SD))	84·2 (9·4)
Hypertension[c], n (%)	21 (52·5)
Daily smoker, n (%)	10 (25·0)

a) *Based on blood glucose and/or use of anti-diabetes medication*
b) *Based on Friedewalds equation:*
   LDL-cholesterol = Total cholesterol – (Triglycerides/2·2) – HDL cholesterol
c) *Based on systolic blood pressure ≥140 or diastolic blood pressure ≥90 or self-reported use of blood pressure lowering medication*

Table S3a: CHDMs identified in the single-timepoint usual care group

ID	Gene name	Location	HGVS c	HGVS p	Literature[a]	VAF[b]	Read count[c] variant / total
1	*ASXL1*	chr20:g.31021227_ 31021228	c.1226dupA	p.Lys410Glufs*28	new LoF *ASXL1*	1·02	19/1814
2	*ASXL1*	chr20:g.31021250	c.1249C>T	p.Arg417Ter	exact	24·11	795/3297
3	*ASXL1*	chr20:g.31022277	c.1762C>T	p.Gln588Ter	exact	6·03	136/2256
4	*ASXL1*	chr20:g.31022288	c.1773C>A	p.Tyr591Ter	exact	4·50	123/2735
5	*ASXL1*	chr20:g.31022592	c.2077C>T	p.Arg693Ter	exact	6·44	184/2858
6	*ASXL1*	chr20:g.31022592	c.2077C>T	p.Arg693Ter	exact	0·55	26/4731
7	*CBL*	chr11:g.119148964	c.1184C>T	p.Pro395Leu	new	6·32	76/1203
7	*DNMT3A*	chr2:g.25463190	c.2303A>G	p.Asp768Gly	overlapping	0·23	18/7965
8	*CBL*	chr11:g.119148991	c.1211G>A	p.Cys404Tyr	exact	0·46	20/4314
8	*DNMT3A*	chr2:g.25464538	c.1975C>G	p.Arg659Gly	exact	1·40	77/5485
9	*CBL*	chr11:g.119148991	c.1211G>A	p.Cys404Tyr	exact	0·29	11/3760
9	*GNAS*	chr20:g.57484417	c.2527T>C	p.Cys843Arg	new	0·34	15/4476
10	*CBL*	chr11:g.119148991	c.1211G>A	p.Cys404Tyr	exact	11·88	354/2979
11	*CBL*	chr11:g.119149241	c.1249C>G	p.Pro417Ala	exact	1·17	54/4606
12	*CBL*	chr11:g.119149250	c.1258C>T	p.Arg420Ter	overlapping	0·03	3/9573
12	*IDH2*	chr15:g.90631934	c.419G>A	p.Arg140Gln	exact	0·06	6/9489
13	*CBL*	chr11:g.119149250	c.1258C>T	p.Arg420Ter	overlapping	0·01	1/9596
13	*GNB1*	chr1:g.1747229	c.169A>G	p.Lys57Glu	exact	1·40	133/9501
14	*CBL*	chr11:g.119149251	c.1259G>A	p.Arg420Gln	exact	24·57	585/2381
15	*CBL*	chr11:g.119149251	c.1259G>A	p.Arg420Gln	exact	0·16	12/7728
16	*DNMT3A*	chr2:g.25457155	c.2732G>A	p.Cys911Tyr	exact	1·28	19/1481
17	*DNMT3A*	chr2:g.25457158	c.2729C>T	p.Ala910Val	exact	0·28	12/4246
17	*SF3B1*	chr2:g.198266834	c.2098A>G	p.Lys700Glu	exact	0·30	29/9708
18	*DNMT3A*	chr2:g.25457158	c.2729C>T	p.Ala910Val	exact	3·04	58/1909
19	*DNMT3A*	chr2:g.25457165	c.2722T>G	p.Tyr908Asp	exact	5·58	281/5032
19	*IDH2*	chr15:g.90631934	c.419G>A	p.Arg140Gln	exact	1·90	82/4321
20	*DNMT3A*	chr2:g.25463286	c.2207G>A	p.Arg736His	exact	0·66	32/4868
20	*DNMT3A*	chr2:g.25457242	c.2645G>A	p.Arg882His	exact	0·52	38/7344

20	*DNMT3A*	chr2:g.25457171	c.2716A>T	p.Lys906Ter	new LoF *DNMT3A*	0·34	22/6523
21	*DNMT3A*	chr2:g.25457171	c.2716A>T	p.Lys906Ter	new LoF *DNMT3A*	2·23	114/5102
22	*DNMT3A*	chr2:g.25457176	c.2711C>T	p.Pro904Leu	exact	0·65	29/4481
23	*DNMT3A*	chr2:g.25457176	c.2711C>T	p.Pro904Leu	exact	0·18	9/4956
24	*DNMT3A*	chr2:g.25457176	c.2711C>T	p.Pro904Leu	exact	0·16	12/7482
25	*DNMT3A*	chr2:g.25457179_ 25457182	c.2705_2708delinsCGG	p.Phe902Serfs*4	exact	2·06	89/4310
26	*DNMT3A*	chr2:g.25463289	c.2204A>G	p.Tyr735Cys	exact	0·19	8/4206
26	*DNMT3A*	chr2:g.25457192	c.2695C>T	p.Arg899Cys	exact	0·19	8/4270
27	*DNMT3A*	chr2:g.25457231	c.2656C>T	p.Gln886Ter	exact	0·22	9/4142
27	*TP53*	chr17:g.7578410	c.520A>G	p.Arg174Gly	new	0·19	11/5671
28	*DNMT3A*	chr2:g.25467433	c.1643T>C	p.Met548Thr	exact	3·28	88/2684
28	*DNMT3A*	chr2:g.25457235	c.2652delG	p.Arg885Glyfs*21	new LoF *DNMT3A*	1·05	43/4076
28	*DNMT3A*	chr2:g.25470498	c.976C>T	p.Arg326Cys	exact	0·52	22/4249
29	*DNMT3A*	chr2:g.25469525	c.1243C>T	p.Gln415Ter	exact	4·54	58/1277
29	*DNMT3A*	chr2:g.25463286	c.2207G>A	p.Arg736His	exact	1·40	33/2362
29	*DNMT3A*	chr2:g.25463184	c.2309C>T	p.Ser770Leu	exact	3·10	223/7187
29	*DNMT3A*	chr2:g.25457242	c.2645G>A	p.Arg882His	exact	0·42	16/3812
30	*DNMT3A*	chr2:g.25470011	c.1031T>C	p.Leu344Pro	exact	1·59	49/3087
30	*DNMT3A*	chr2:g.25463190	c.2303A>G	p.Asp768Gly	overlapping	0·03	3/9827
30	*DNMT3A*	chr2:g.25457249	c.2638A>G	p.Met880Val	exact	0·17	11/6545
30	*DNMT3A*	chr2:g.25457242	c.2645G>A	p.Arg882His	exact	0·41	27/6508
31	*DNMT3A*	chr2:g.25466767	c.1936G>A	p.Gly646Arg	exact	1·75	54/3079
31	*DNMT3A*	chr2:g.25463308	c.2185C>T	p.Arg729Trp	exact	1·04	51/4886
31	*DNMT3A*	chr2:g.25457242	c.2645G>A	p.Arg882His	exact	0·43	17/3972
32	*DNMT3A*	chr2:g.25463182	c.2311C>T	p.Arg771Ter	exact	9·49	570/6005
32	*DNMT3A*	chr2:g.25457242	c.2645G>A	p.Arg882His	exact	3·75	120/3202
33	*DNMT3A*	chr2:g.25457242	c.2645G>A	p.Arg882His	exact	23·04	1419/6160
34	*DNMT3A*	chr2:g.25457242	c.2645G>A	p.Arg882His	exact	13·06	324/2481
35	*DNMT3A*	chr2:g.25457242	c.2645G>A	p.Arg882His	exact	0·90	40/4444

36	*DNMT3A*	chr2:g.25457242	c.2645G>A	p.Arg882His	exact	0·77	33/4292
37	*DNMT3A*	chr2:g.25457242	c.2645G>A	p.Arg882His	exact	0·55	30/5446
38	*DNMT3A*	chr2:g.25457242	c.2645G>A	p.Arg882His	exact	0·42	18/4337
39	*DNMT3A*	chr2:g.25466779	c.1924G>A	p.Gly642Arg	overlapping	3·75	207/5525
39	*DNMT3A*	chr2:g.25457243	c.2644C>T	p.Arg882Cys	exact	0·76	43/5638
39	*SF3B1*	chr2:g.198266834	c.2098A>G	p.Lys700Glu	exact	0·20	12/6129
40	*DNMT3A*	chr2:g.25462078	c.2329C>G	p.Pro777Ala	overlapping	0·60	44/7278
40	*DNMT3A*	chr2:g.25457243	c.2644C>A	p.Arg882Ser	exact	0·87	45/5143
41	*DNMT3A*	chr2:g.25469614	c.1154delC	p.Pro385Argfs*22	exact	4·71	36/765
41	*DNMT3A*	chr2:g.25457243	c.2644C>T	p.Arg882Cys	exact	0·32	14/4398
42	*DNMT3A*	chr2:g.25457243	c.2644C>T	p.Arg882Cys	exact	21·25	797/3750
43	*DNMT3A*	chr2:g.25457243	c.2644C>T	p.Arg882Cys	exact	7·21	237/3288
44	*DNMT3A*	chr2:g.25457243	c.2644C>T	p.Arg882Cys	exact	1·93	57/2951
45	*DNMT3A*	chr2:g.25457243	c.2644C>T	p.Arg882Cys	exact	0·64	21/3266
46	*DNMT3A*	chr2:g.25457243	c.2644C>A	p.Arg882Ser	exact	0·57	17/2959
47	*DNMT3A*	chr2:g.25457243	c.2644C>T	p.Arg882Cys	exact	0·56	29/5197
48	*DNMT3A*	chr2:g.25457243	c.2644C>T	p.Arg882Cys	exact	0·23	11/4855
49	*DNMT3A*	chr2:g.25457249	c.2638A>G	p.Met880Val	exact	0·39	13/3332
50	*DNMT3A*	chr2:g.25457249	c.2638A>G	p.Met880Val	exact	0·18	12/6605
51	*DNMT3A*	chr2:g.25457255	c.2632T>C	p.Ser878Pro	exact	1·46	58/3986
52	*DNMT3A*	chr2:g.25457269	c.2618A>G	p.His873Arg	exact	0·25	10/4054
53	*DNMT3A*	chr2:g.25463297_ 25463300	c.2193_2196delCTTT	p.Phe731Leufs*47	exact	0·86	35/4082
53	*DNMT3A*	chr2:g.25458595	c.2578T>C	p.Trp860Arg	exact	0·20	19/9634
54	*DNMT3A*	chr2:g.25458595	c.2578T>C	p.Trp860Arg	exact	0·91	89/9791
55	*DNMT3A*	chr2:g.25463302	c.2191T>C	p.Phe731Leu	exact	0·23	12/5223
55	*DNMT3A*	chr2:g.25458595_ 25458596	c.2577dupA	p.Trp860Metfs*4	exact	0·55	41/7507
56	*DNMT3A*	chr2:g.25458595_ 25458596	c.2577dupA	p.Trp860Metfs*4	exact	0·34	21/6158
57	*DNMT3A*	chr2:g.25463181	c.2312G>A	p.Arg771Gln	exact	0·53	52/9837
57	*DNMT3A*	chr2:g.25458604	c.2569G>T	p.Asp857Tyr	overlapping	0·73	50/6860
58	*DNMT3A*	chr2:g.25458656	c.2517delC	p.Ile840Ter	exact	9·78	382/3905

59	*DNMT3A*	chr2:g.25458684	c.2489T>C	p.Val830Ala	overlapping	1·16	61/5261
59	*GNAS*	chr20:g.57484417	c.2527T>C	p.Cys843Arg	new	0·22	8/3648
60	*DNMT3A*	chr2:g.25463586	c.2096G>A	p.Gly699Asp	exact	11·36	350/3081
60	*DNMT3A*	chr2:g.25459806	c.2477A>C	p.Lys826Thr	exact	2·27	132/5816
60	*KRAS*	chr12:g.25398279	c.40G>A	p.Val14Ile	exact	4·70	163/3470
61	*DNMT3A*	chr2:g.25463247	c.2246G>A	p.Arg749His	exact	7·56	43/569
61	*DNMT3A*	chr2:g.25462012	c.2395C>T	p.Pro799Ser	exact	6·61	189/2858
62	*DNMT3A*	chr2:g.25467448	c.1628G>C	p.Gly543Ala	exact	0·85	21/2472
62	*DNMT3A*	chr2:g.25462020	c.2387G>A	p.Gly796Asp	exact	2·47	47/1902
63	*DNMT3A*	chr2:g.25462035	c.2372C>T	p.Ala791Val	exact	23·25	443/1905
64	*DNMT3A*	chr2:g.25463190	c.2303A>G	p.Asp768Gly	overlapping	0·02	2/9829
64	*DNMT3A*	chr2:g.25462068	c.2339T>C	p.Ile780Thr	exact	0·28	18/6522
64	*GNAS*	chr20:g.57484417	c.2527T>C	p.Cys843Arg	new	0·17	10/5880
64	*IDH2*	chr15:g.90631934	c.419G>A	p.Arg140Gln	exact	1·04	69/6608
65	*DNMT3A*	chr2:g.25462068	c.2339T>C	p.Ile780Thr	exact	0·51	17/3363
65	*GNB1*	chr1:g.1747229	c.169A>G	p.Lys57Glu	exact	3·23	191/5917
66	*DNMT3A*	chr2:g.25463289	c.2204A>G	p.Tyr735Cys	exact	0·70	19/2724
66	*DNMT3A*	chr2:g.25462068	c.2339T>C	p.Ile780Thr	exact	0·36	14/3913
67	*DNMT3A*	chr2:g.25463190	c.2303A>G	p.Asp768Gly	overlapping	0·20	20/9857
67	*DNMT3A*	chr2:g.25462068	c.2339T>C	p.Ile780Thr	exact	0·17	9/5352
68	*DNMT3A*	chr2:g.25462068	c.2339T>C	p.Ile780Thr	exact	0·36	12/3308
69	*DNMT3A*	chr2:g.25462074	c.2333T>G	p.Val778Gly	overlapping	0·40	13/3272
70	*DNMT3A*	chr2:g.25463307	c.2186G>T	p.Arg729Leu	exact	4·02	296/7364
70	*DNMT3A*	chr2:g.25462075	c.2332delG	p.Val778Ter	exact	1·58	90/5713
71	*DNMT3A*	chr2:g.25464537	c.1976G>A	p.Arg659His	exact	0·38	10/2664
71	*DNMT3A*	chr2:g.25462077	c.2330C>T	p.Pro777Leu	exact	0·59	22/3758
72	*DNMT3A*	chr2:g.25462077	c.2330C>T	p.Pro777Leu	exact	0·52	13/2489
73	*DNMT3A*	chr2:g.25462078	c.2329C>G	p.Pro777Ala	overlapping	0·26	15/5846
74	*DNMT3A*	chr2:g.25463173	c.2320G>A	p.Glu774Lys	exact	19·22	754/3922
75	*DNMT3A*	chr2:g.25463179	c.2314T>A	p.Phe772Ile	overlapping	0·59	54/9163
76	*DNMT3A*	chr2:g.25463181	c.2312G>C	p.Arg771Pro	exact	0·72	64/8849
77	*DNMT3A*	chr2:g.25463181	c.2312G>A	p.Arg771Gln	exact	0·13	13/9802

78	*DNMT3A*	chr2:g.25464570	c.1943T>C	p.Leu648Pro	new	0·13	10/7905
78	*DNMT3A*	chr2:g.25463182	c.2311C>T	p.Arg771Ter	exact	0·20	20/9781
78	*TP53*	chr17:g.7578410	c.520A>G	p.Arg174Gly	new	0·18	17/9298
79	*DNMT3A*	chr2:g.25463182	c.2311C>T	p.Arg771Ter	exact	0·83	82/9824
80	*DNMT3A*	chr2:g.25463182	c.2311C>T	p.Arg771Ter	exact	0·64	50/7757
81	*DNMT3A*	chr2:g.25463182	c.2311C>T	p.Arg771Ter	exact	0·37	22/5887
82	*DNMT3A*	chr2:g.25463182	c.2311C>T	p.Arg771Ter	exact	0·33	28/8583
83	*DNMT3A*	chr2:g.25463299	c.2194T>A	p.Phe732Ile	exact	7·10	501/7052
83	*DNMT3A*	chr2:g.25463184	c.2309C>T	p.Ser770Leu	exact	0·20	20/9805
83	*TET2*	chr4:g.106157653	c.2617G>T	p.Glu873Ter	new LoF *TET2*	7·15	197/2754
84	*DNMT3A*	chr2:g.25466800	c.1903C>T	p.Arg635Trp	exact	2·11	87/4114
84	*DNMT3A*	chr2:g.25463184	c.2309C>T	p.Ser770Leu	exact	0·21	20/9606
85	*DNMT3A*	chr2:g.25463184	c.2309C>T	p.Ser770Leu	exact	2·21	185/8378
86	*DNMT3A*	chr2:g.25463184	c.2309C>T	p.Ser770Leu	exact	0·14	13/9229
87	*DNMT3A*	chr2:g.25463186	c.2307C>G	p.Ile769Met	overlapping	1·78	149/8357
88	*DNMT3A*	chr2:g.25463190	c.2303A>T	p.Asp768Val	overlapping	2·22	213/9581
89	*DNMT3A*	chr2:g.25463190	c.2303A>G	p.Asp768Gly	overlapping	0·15	15/9817
90	*DNMT3A*	chr2:g.25463190	c.2303A>G	p.Asp768Gly	overlapping	0·14	14/9811
91	*DNMT3A*	chr2:g.25463190	c.2303A>G	p.Asp768Gly	overlapping	0·14	14/9772
92	*DNMT3A*	chr2:g.25463190	c.2303A>G	p.Asp768Gly	overlapping	0·11	11/9851
93	*DNMT3A*	chr2:g.25463190	c.2303A>G	p.Asp768Gly	overlapping	0·03	3/9617
94	*DNMT3A*	chr2:g.25463229	c.2264T>C	p.Phe755Ser	exact	1·06	47/4449
95	*DNMT3A*	chr2:g.25463229	c.2264T>C	p.Phe755Ser	exact	0·39	20/5068
96	*DNMT3A*	chr2:g.25463229	c.2264T>C	p.Phe755Ser	exact	0·30	10/3350
97	*DNMT3A*	chr2:g.25463308	c.2185C>T	p.Arg729Trp	exact	0·44	32/7234
97	*DNMT3A*	chr2:g.25463230	c.2263T>A	p.Phe755Ile	exact	1·81	108/5982
98	*DNMT3A*	chr2:g.25463235	c.2258G>A	p.Trp753Ter	exact	1·65	60/3645
99	*DNMT3A*	chr2:g.25463283	c.2210T>G	p.Leu737Arg	exact	0·86	58/6746
100	*DNMT3A*	chr2:g.25463284_ 25463285	c.2208_2209insATCACTTTGAGTTCTACCGC	p.Leu737Ilefs*49	exact	1·38	39/2786
101	*DNMT3A*	chr2:g.25463286	c.2207G>A	p.Arg736His	exact	4·10	173/4215

102	*DNMT3A*	chr2:g.25463286	c.2207G>A	p.Arg736His	exact	0·67	22/3308
103	*DNMT3A*	chr2:g.25463286	c.2207G>A	p.Arg736His	exact	0·63	16/2524
104<br>104	*DNMT3A*<br>*DNMT3A*	chr2:g.25467083<br>chr2:g.25463287	c.1792C>T<br>c.2206C>G	p.Arg598Ter<br>p.Arg736Gly	exact<br>exact	0·60<br>1·29	14/2337<br>32/2476
105<br>105	*DNMT3A*<br>*DNMT3A*	chr2:g.25464544<br>chr2:g.25463287	c.1969G>A<br>c.2206C>T	p.Val657Met<br>p.Arg736Cys	exact<br>exact	1·33<br>1·04	40/3009<br>22/2107
106	*DNMT3A*	chr2:g.25463287	c.2206C>T	p.Arg736Cys	exact	24·02	955/3976
107	*DNMT3A*	chr2:g.25463287	c.2206C>T	p.Arg736Cys	exact	10·29	174/1691
108	*DNMT3A*	chr2:g.25463287	c.2206C>T	p.Arg736Cys	exact	0·65	23/3558
109	*DNMT3A*	chr2:g.25463287	c.2206C>T	p.Arg736Cys	exact	0·61	21/3463
110	*DNMT3A*	chr2:g.25463287	c.2206C>G	p.Arg736Gly	exact	0·37	14/3739
111<br>111	*DNMT3A*<br>*DNMT3A*	chr2:g.25463289<br>chr2:g.25470480	c.2204A>G<br>c.994G>A	p.Tyr735Cys<br>p.Gly332Arg	exact<br>exact	0·71<br>0·53	16/2251<br>16/3020
112	*DNMT3A*	chr2:g.25463289	c.2204A>C	p.Tyr735Ser	exact	1·39	23/1660
113	*DNMT3A*	chr2:g.25463289	c.2204A>G	p.Tyr735Cys	exact	1·38	31/2254
114	*DNMT3A*	chr2:g.25463289	c.2204A>G	p.Tyr735Cys	exact	0·94	31/3288
115	*DNMT3A*	chr2:g.25463289	c.2204A>G	p.Tyr735Cys	exact	0·56	16/2840
116	*DNMT3A*	chr2:g.25463289	c.2204A>G	p.Tyr735Cys	exact	0·49	14/2837
117	*DNMT3A*	chr2:g.25463289	c.2204A>G	p.Tyr735Cys	exact	0·40	9/2256
118	*DNMT3A*	chr2:g.25463289	c.2204A>G	p.Tyr735Cys	exact	0·27	11/4058
119	*DNMT3A*	chr2:g.25463296	c.2197G>T	p.Glu733Ter	exact	2·18	111/5083
120<br>120	*DNMT3A*<br>*SF3B1*	chr2:g.25463296_ 25463297<br>chr2:g.198266834	c.2196dupT<br>c.2098A>G	p.Glu733Ter<br>p.Lys700Glu	exact<br>exact	31·15<br>3·33	261/838<br>290/8719
121	*DNMT3A*	chr2:g.25463296_ 25463297	c.2196dupT	p.Glu733Ter	exact	0·23	8/3485
122	*DNMT3A*	chr2:g.25463298_ 25463300	c.2193_2195delCTT	p.Phe732del	exact	13·21	582/4405
123	*DNMT3A*	chr2:g.25463299_ 25463300	c.2193_2194delCT	p.Phe732Ter	exact	5·95	489/8224
124	*DNMT3A*	chr2:g.25463307	c.2186G>A	p.Arg729Gln	exact	0·88	60/6800
125	*DNMT3A*	chr2:g.25463308	c.2185C>T	p.Arg729Trp	exact	6·33	264/4171
126	*DNMT3A*	chr2:g.25463308	c.2185C>T	p.Arg729Trp	exact	1·10	49/4469

127	*DNMT3A*	chr2:g.25463308	c.2185C>T	p.Arg729Trp	exact	0·58	24/4146
128	*DNMT3A*	chr2:g.25463308	c.2185C>T	p.Arg729Trp	exact	0·47	31/6556
129	*DNMT3A*	chr2:g.25463308	c.2185C>T	p.Arg729Trp	exact	0·45	29/6450
130	*DNMT3A*	chr2:g.25463308	c.2185C>G	p.Arg729Gly	exact	0·40	22/5494
131	*DNMT3A*	chr2:g.25463308	c.2185C>T	p.Arg729Trp	exact	0·28	21/7616
132	*DNMT3A*	chr2:g.25463308	c.2185C>T	p.Arg729Trp	exact	0·14	9/6544
133	*DNMT3A*	chr2:g.25463316	c.2177G>T	p.Gly726Val	exact	2·09	49/2348
134	*DNMT3A*	chr2:g.25463536_ 25463537	c.2145_2146insTT	p.Val716Leufs*64	exact	12·94	147/1136
135	*DNMT3A*	chr2:g.25463541	c.2141C>G	p.Ser714Cys	exact	1·55	37/2394
136	*DNMT3A*	chr2:g.25463577	c.2105A>G	p.Asp702Gly	overlapping	0·90	32/3569
137	*DNMT3A*	chr2:g.25463583	c.2099C>T	p.Pro700Leu	exact	0·53	10/1880
138	*DNMT3A*	chr2:g.25463584	c.2098C>A	p.Pro700Thr	exact	1·69	34/2014
139	*DNMT3A*	chr2:g.25463588	c.2094G>A	p.Trp698Ter	exact	10·14	168/1656
140	*DNMT3A*	chr2:g.25464444	c.2069T>A	p.Val690Asp	exact	26·46	326/1232
141	*DNMT3A*	chr2:g.25464444	c.2069T>A	p.Val690Asp	exact	17·11	468/2735
142	*DNMT3A*	chr2:g.25464534	c.1979A>G	p.Tyr660Cys	exact	0·29	15/5137
143	*DNMT3A*	chr2:g.25464537	c.1976G>A	p.Arg659His	exact	0·79	43/5414
144	*DNMT3A*	chr2:g.25464540_ 25464541	c.1970_1972dupTGG	p.Val657dup	overlapping	2·22	89/4009
145	*DNMT3A*	chr2:g.25468159	c.1517A>G	p.His506Arg	exact	8·78	102/1162
145	*DNMT3A*	chr2:g.25464544	c.1969G>A	p.Val657Met	exact	1·57	45/2858
146	*DNMT3A*	chr2:g.25464544	c.1969G>A	p.Val657Met	exact	1·40	24/1719
146	*TET2*	chr4:g.106156704_ 106156707	c.1668_1671delCAAA	p.Asn556Lysfs*6	exact	0·68	30/4321
147	*DNMT3A*	chr2:g.25464554	c.1959G>C	p.Leu653Phe	exact	1·24	42/3394
148	*DNMT3A*	chr2:g.25464574_ 25464575	c.1938dupG	p.Leu647Alafs*21	exact	1·22	116/9530
149	*DNMT3A*	chr2:g.25464575	c.1938delG	p.Leu647Serfs*4	exact	0·69	32/4651
150	*DNMT3A*	chr2:g.25466779	c.1924G>A	p.Gly642Arg	overlapping	7·30	237/3248
151	*DNMT3A*	chr2:g.25470019	c.1023delT	p.Glu342Argfs*3	new LoF *DNMT3A*	11·89	361/3037
151	*DNMT3A*	chr2:g.25466799	c.1904G>T	p.Arg635Leu	exact	3·68	126/3424

152	*DNMT3A*	chr2:g.25466799	c.1904G>A	p.Arg635Gln	exact	0·51	16/3142
153<br>153	*DNMT3A*<br>*JAK2*	chr2:g.25466800<br>chr9:g.5073770	c.1903C>T<br>c.1849G>T	p.Arg635Trp<br>p.Val617Phe	exact<br>exact	0·57<br>0·17	15/2640<br>10/5727
154	*DNMT3A*	chr2:g.25466800	c.1903C>T	p.Arg635Trp	exact	0·49	15/3060
155	*DNMT3A*	chr2:g.25467083	c.1792C>T	p.Arg598Ter	exact	1·66	56/3372
156	*DNMT3A*	chr2:g.25467091	c.1784T>C	p.Leu595Pro	exact	10·56	386/3655
157	*DNMT3A*	chr2:g.25467200	c.1675delT	p.Cys559Alafs*92	exact	0·43	5/1156
158	*DNMT3A*	chr2:g.25467434	c.1642A>G	p.Met548Val	overlapping	2·00	32/1599
159	*DNMT3A*	chr2:g.25467442	c.1634A>G	p.Glu545Gly	overlapping	2·07	49/2365
160	*DNMT3A*	chr2:g.25467449	c.1627G>T	p.Gly543Cys	exact	5·98	75/1255
161	*DNMT3A*	chr2:g.25469932_ 25469933	c.1109dupA	p.Tyr370Ter	exact	0·67	18/2683
162	*DNMT3A*	chr2:g.25469945	c.1097G>C	p.Arg366Pro	overlapping	7·97	66/828
163	*DNMT3A*	chr2:g.25470011	c.1031T>C	p.Leu344Pro	exact	19·73	575/2915
164	*DNMT3A*	chr2:g.25470011	c.1031T>A	p.Leu344Gln	exact	1·84	42/2278
165	*DNMT3A*	chr2:g.25470011	c.1031T>C	p.Leu344Pro	exact	0·55	11/2001
166	*DNMT3A*	chr2:g.25470480	c.994G>A	p.Gly332Arg	exact	0·44	15/3438
167	*DNMT3A*	chr2:g.25470483	c.991T>A	p.Phe331Ile	overlapping	1·65	42/2552
168<br>168	*DNMT3A*<br>*DNMT3A*	chr2:g.25470497<br>chr2:g.25470484	c.977G>A<br>c.990G>T	p.Arg326His<br>p.Trp330Cys	exact<br>overlapping	0·41<br>0·37	17/4126<br>16/4371
169	*DNMT3A*	chr2:g.25470497	c.977G>A	p.Arg326His	exact	0·94	27/2865
170	*DNMT3A*	chr2:g.25470497	c.977G>A	p.Arg326His	exact	0·33	9/2709
171	*DNMT3A*	chr2:g.25470498	c.976C>T	p.Arg326Cys	exact	11·78	486/4125
172	*DNMT3A*	chr2:g.25470498	c.976C>T	p.Arg326Cys	exact	1·24	44/3542
173	*DNMT3A*	chr2:g.25470516	c.958C>T	p.Arg320Ter	exact	2·09	118/5646
174	*DNMT3A*	chr2:g.25470533	c.941G>A	p.Trp314Ter	exact	2·96	18/608
175	*GNAS*	chr20:g.57484414	c.2524C>T	p.Arg842Cys	new	0·07	6/9114
176	*GNAS*	chr20:g.57484414	c.2524C>T	p.Arg842Cys	new	0·02	2/9727
177	*GNAS*	chr20:g.57484417	c.2527T>C	p.Cys843Arg	new	0·45	19/4244
178	*GNAS*	chr20:g.57484417	c.2527T>C	p.Cys843Arg	new	0·37	12/3226
179	*GNAS*	chr20:g.57484417	c.2527T>C	p.Cys843Arg	new	0·29	12/4124
180	*GNAS*	chr20:g.57484417	c.2527T>C	p.Cys843Arg	new	0·24	19/7862

181	*GNAS*	chr20:g.57484417	c.2527T>C	p.Cys843Arg	new	0·22	13/5841
182	*GNAS*	chr20:g.57484417	c.2527T>C	p.Cys843Arg	new	0·13	9/6918
183	*GNAS*	chr20:g.57484420	c.2530C>T	p.Arg844Cys	exact	0·29	14/4815
184	*GNAS*	chr20:g.57484421	c.2531G>A	p.Arg844His	exact	1·99	84/4224
185	*GNAS*	chr20:g.57484421	c.2531G>A	p.Arg844His	exact	1·90	92/4834
186	*GNAS*	chr20:g.57484421	c.2531G>A	p.Arg844His	exact	1·38	54/3904
187	*GNAS*	chr20:g.57484421	c.2531G>A	p.Arg844His	exact	0·94	20/2134
188	*GNAS*	chr20:g.57484421	c.2531G>A	p.Arg844His	exact	0·31	12/3812
189	*GNAS*	chr20:g.57484421	c.2531G>A	p.Arg844His	exact	0·23	22/9712
190	*GNAS*	chr20:g.57484421	c.2531G>A	p.Arg844His	exact	0·21	10/4754
191	*GNB1*	chr1:g.1747227	c.171G>T	p.Lys57Asn	exact	0·26	10/3827
192	*GNB1*	chr1:g.1747229	c.169A>G	p.Lys57Glu	exact	3·43	90/2627
193	*GNB1*	chr1:g.1747229	c.169A>G	p.Lys57Glu	exact	1·68	89/5302
194	*GNB1*	chr1:g.1747229	c.169A>G	p.Lys57Glu	exact	0·70	56/7959
195	*GNB1*	chr1:g.1747229	c.169A>G	p.Lys57Glu	exact	0·70	48/6833
196	*GNB1*	chr1:g.1747229	c.169A>G	p.Lys57Glu	exact	0·62	43/6990
197	*IDH2*	chr15:g.90631934	c.419G>A	p.Arg140Gln	exact	0·18	8/4548
198	*IDH2*	chr15:g.90631935	c.418C>T	p.Arg140Trp	exact	0·10	9/9291
199	*JAK2*	chr9:g.5073770	c.1849G>T	p.Val617Phe	exact	13·69	326/2382
200	*JAK2*	chr9:g.5073770	c.1849G>T	p.Val617Phe	exact	6·78	153/2257
201	*JAK2*	chr9:g.5073770	c.1849G>T	p.Val617Phe	exact	0·94	17/1810
202	*JAK2*	chr9:g.5073770	c.1849G>T	p.Val617Phe	exact	0·54	35/6493
203	*JAK2*	chr9:g.5073770	c.1849G>T	p.Val617Phe	exact	0·19	13/6861
204	*KRAS*	chr12:g.25398281	c.38G>A	p.Gly13Asp	exact	0·92	17/1847
205	*KRAS*	chr12:g.25398284	c.35G>A	p.Gly12Asp	exact	0·33	32/9618
206	*SF3B1*	chr2:g.198266834	c.2098A>G	p.Lys700Glu	exact	5·48	148/2699
207	*STAT3*	chr17:g.40474420	c.1981G>T	p.Asp661Tyr	exact	1·36	44/3230
208	*STAT3*	chr17:g.40474420	c.1981G>T	p.Asp661Tyr	exact	0·32	14/4391
209	*TET2*	chr4:g.106156704_ 106156707	c.1668_1671delCAAA	p.Asn556Lysfs*6	exact	0·17	17/9783
210	*TET2*	chr4:g.106156707_ 106156708	c.1671dupA	p.Glu558Argfs*30	new LoF *TET2*	0·92	46/5001

211	*TET2*	chr4:g.106156729	c.1693C>T	p.Arg565Ter	new LoF *TET2*	0·06	6/9466
212	*TET2*	chr4:g.106156747	c.1711C>T	p.Arg571Ter	exact	9·79	207/2114
213	*TP53*	chr17:g.7578410	c.520A>G	p.Arg174Gly	new	0·24	7/2877
214	*TP53*	chr17:g.7578410	c.520A>G	p.Arg174Gly	new	0·17	13/7564
215	*TP53*	chr17:g.7578410	c.520A>G	p.Arg174Gly	new	0·.15	13/8940
216	*TP53*	chr17:g.7578410	c.520A>G	p.Arg174Gly	new	0·13	9/6776

a) *CHDMs in the literature are listed in Table S4a*
b) *We cannot exclude that some detected somatic mutations with very low VAF are false positives*
c) *Average variant and total read counts for PCR1 and PCR2. Generated by mpileups of all non-error-corrected reads; insertions and deletions are manually checked for read counts in the IGV browser*

Table S3b: CHDMs identified in the single-timepoint bariatric surgery group

ID	Gene name	Location	HGVS c	HGVS p	Literature[a]	VAF[b]	Read count[c] variant / total
217	*ASXL1*	chr20:g.31021250	c.1249C>T	p.Arg417Ter	exact	5·53	137/2476
218	*ASXL1*	chr20:g.31022904	c.2389G>T	p.Glu797Ter	overlapping	0·93	48/5176
219	*BRCC3*	chrX:g.154305514	c.265C>T	p.Arg89Ter	exact	3·07	56/1825
219	*GNAS*	chr20:g.57484417	c.2527T>C	p.Cys843Arg	new	0·25	10/3963
220	*BRCC3*	chrX:g.154305522	c.273A>T	p.Glu91Asp	new	14·46	104/719
221	*CBL*	chr11:g.119149003	c.1223G>C	p.Trp408Ser	overlapping	2·88	65/2257
222	*CBL*	chr11:g.119149250	c.1258C>T	p.Arg420Ter	overlapping	0·22	13/5908
223	*CBL*	chr11:g.119149251	c.1259G>A	p.Arg420Gln	exact	1·11	25/2252
224	*CBL*	chr11:g.119149251	c.1259G>A	p.Arg420Gln	exact	3·23	121/3742
225	*CBL*	chr11:g.119149251	c.1259G>A	p.Arg420Gln	exact	0·13	12/9552
226	*DNMT3A*	chr2:g.25463289	c.2204A>G	p.Tyr735Cys	exact	0·55	18/3252
226	*DNMT3A*	chr2:g.25458585	c.2588A>T	p.Glu863Val	exact	0·65	48/7347
226	*DNMT3A*	chr2:g.25457158	c.2729C>T	p.Ala910Val	exact	8·06	275/3413
226	*JAK2*	chr9:g.5073770	c.1849G>T	p.Val617Phe	exact	0·32	18/5549
227	*DNMT3A*	chr2:g.25463308	c.2185C>G	p.Arg729Gly	exact	0·25	17/6829
227	*DNMT3A*	chr2:g.25463182_ 25463183	c.2301_2310dupGGACATCTCG	p.Arg771Glyfs*14	overlapping	1·51	145/9615
227	*DNMT3A*	chr2:g.25457176	c.2711C>T	p.Pro904Leu	exact	0·13	11/8403
228	*DNMT3A*	chr2:g.25457176	c.2711C>T	p.Pro904Leu	exact	0·38	23/6067
228	*TET2*	chr4:g.106157725	c.2689C>T	p.Gln897Ter	overlapping	1·29	35/2706
229	*DNMT3A*	chr2:g.25457176	c.2711C>T	p.Pro904Leu	exact	0·22	10/4532
230	*DNMT3A*	chr2:g.25457176	c.2711C>T	p.Pro904Leu	exact	0·10	8/7867
231	*DNMT3A*	chr2:g.25457176	c.2711C>T	p.Pro904Leu	exact	2·40	144/6010
232	*DNMT3A*	chr2:g.25457176	c.2711C>A	p.Pro904Gln	exact	1·44	100/6961
233	*DNMT3A*	chr2:g.25457176	c.2711C>T	p.Pro904Leu	exact	1·02	22/2156
234	*DNMT3A*	chr2:g.25462074	c.2333T>G	p.Val778Gly	overlapping	0·71	37/5210
234	*DNMT3A*	chr2:g.25457186	c.2701C>G	p.Leu901Val	exact	1·86	72/3879
234	*TP53*	chr17:g.7578410	c.520A>G	p.Arg174Gly	new	0·15	10/6515
235	*DNMT3A*	chr2:g.25457192	c.2695C>T	p.Arg899Cys	exact	4·46	108/2419

236	*DNMT3A*	chr2:g.25457192	c.2695C>T	p.Arg899Cys	exact	0·60	18/3015
237	*DNMT3A*	chr2:g.25457212	c.2675C>G	p.Ser892Ter	new	1·00	51/5103
238	*DNMT3A*	chr2:g.25467190	c.1685G>T	p.Cys562Phe	overlapping	6·03	192/3184
238	*DNMT3A*	chr2:g.25463588	c.2094G>A	p.Trp698Ter	exact	1·02	34/3349
238	*DNMT3A*	chr2:g.25457242	c.2645G>A	p.Arg882His	exact	3·34	195/5845
239	*DNMT3A*	chr2:g.25457242	c.2645G>A	p.Arg882His	exact	1·01	34/3366
240	*DNMT3A*	chr2:g.25457242	c.2645G>A	p.Arg882His	exact	0·84	29/3472
241	*DNMT3A*	chr2:g.25457242	c.2645G>A	p.Arg882His	exact	2·43	98/4037
242	*DNMT3A*	chr2:g.25457242	c.2645G>A	p.Arg882His	exact	1·63	37/2272
243	*DNMT3A*	chr2:g.25457242	c.2645G>A	p.Arg882His	exact	12·91	550/4259
244	*DNMT3A*	chr2:g.25457242	c.2645G>A	p.Arg882His	exact	1·88	63/3351
245	*DNMT3A*	chr2:g.25457242	c.2645G>A	p.Arg882His	exact	1·16	70/6043
246	*DNMT3A*	chr2:g.25457242	c.2645G>A	p.Arg882His	exact	0·21	10/4797
247	*DNMT3A*	chr2:g.25468910	c.1453C>T	p.Gln485Ter	exact	0·61	30/4911
247	*DNMT3A*	chr2:g.25464533	c.1980C>A	p.Tyr660Ter	overlapping	2·35	89/3784
247	*DNMT3A*	chr2:g.25457243	c.2644C>A	p.Arg882Ser	exact	0·88	39/4455
248	*DNMT3A*	chr2:g.25457243	c.2644C>T	p.Arg882Cys	exact	0·48	14/2928
248	*DNMT3A*	chr2:g.25470480	c.994G>A	p.Gly332Arg	exact	0·63	17/2685
249	*DNMT3A*	chr2:g.25464544	c.1969G>A	p.Val657Met	exact	0·50	15/2982
249	*DNMT3A*	chr2:g.25457243	c.2644C>T	p.Arg882Cys	exact	0·82	29/3531
250	*DNMT3A*	chr2:g.25463182	c.2311C>T	p.Arg771Ter	exact	4·82	325/6747
250	*DNMT3A*	chr2:g.25457243	c.2644C>T	p.Arg882Cys	exact	1·45	48/3321
251	*DNMT3A*	chr2:g.25457243	c.2644C>A	p.Arg882Ser	exact	3·35	124/3698
252	*DNMT3A*	chr2:g.25457243	c.2644C>T	p.Arg882Cys	exact	0·69	15/2172
253	*DNMT3A*	chr2:g.25457243	c.2644C>T	p.Arg882Cys	exact	2·07	76/3664
254	*DNMT3A*	chr2:g.25457243	c.2644C>T	p.Arg882Cys	exact	0·46	26/5714
255	*DNMT3A*	chr2:g.25457243	c.2644C>T	p.Arg882Cys	exact	2·91	48/1651
256	*DNMT3A*	chr2:g.25457243	c.2644C>T	p.Arg882Cys	exact	0·33	13/3899
257	*DNMT3A*	chr2:g.25457249	c.2638A>G	p.Met880Val	exact	0·44	23/5278
258	*DNMT3A*	chr2:g.25457249	c.2638A>G	p.Met880Val	exact	0·33	11/3381
259	*DNMT3A*	chr2:g.25457269	c.2618A>G	p.His873Arg	exact	6·20	335/5401

260	*DNMT3A*	chr2:g.25457269	c.2618A>G	p.His873Arg	exact	0·28	10/3632
261	*DNMT3A*	chr2:g.25457288	c.2599delG	p.Val867Tyrfs*14	exact	22·39	543/2425
262	*DNMT3A*	chr2:g.25458595	c.2578T>C	p.Trp860Arg	exact	1·12	89/7935
263	*DNMT3A*	chr2:g.25458595	c.2578T>C	p.Trp860Arg	exact	1·96	127/6466
264	*DNMT3A*	chr2:g.25458595_ 25458596	c.2577dupA	p.Trp860Metfs*4	exact	0·37	22/5887
265	*DNMT3A*	chr2:g.25463286	c.2207G>A	p.Arg736His	exact	6·79	111/1634
265	*DNMT3A*	chr2:g.25459837	c.2446C>T	p.Gln816Ter	exact	2·93	49/1675
265	*DNMT3A*	chr2:g.25458652	c.2521A>T	p.Lys841Ter	exact	1·18	45/3823
266	*DNMT3A*	chr2:g.25458659_ 25458660	c.2513dupA	p.Asn838Lysfs*17	exact	7·63	506/6636
267	*DNMT3A*	chr2:g.25461999	c.2408G>C	p.Arg803Thr	overlapping	10·44	188/1801
268	*DNMT3A*	chr2:g.25462000	c.2407A>T	p.Arg803Trp	exact	9·48	280/2955
269	*DNMT3A*	chr2:g.25462020	c.2387G>A	p.Gly796Asp	exact	6·18	84/1360
270	*DNMT3A*	chr2:g.25466800	c.1903C>T	p.Arg635Trp	exact	1·30	54/4156
270	*DNMT3A*	chr2:g.25462068	c.2339T>C	p.Ile780Thr	exact	1·94	76/3916
271	*DNMT3A*	chr2:g.25462068	c.2339T>C	p.Ile780Thr	exact	0·25	10/4029
272	*DNMT3A*	chr2:g.25462068	c.2339T>C	p.Ile780Thr	exact	0·79	29/3685
273	*DNMT3A*	chr2:g.25462068	c.2339T>C	p.Ile780Thr	exact	5·47	249/4550
274	*DNMT3A*	chr2:g.25462068	c.2339T>C	p.Ile780Thr	exact	0·32	15/4682
275	*DNMT3A*	chr2:g.25462074	c.2333T>A	p.Val778Glu	overlapping	2·91	98/3362
276	*DNMT3A*	chr2:g.25463172	c.2321A>G	p.Glu774Gly	exact	0·99	42/4237
277	*DNMT3A*	chr2:g.25463180	c.2313delA	p.Leu773Serfs*6	overlapping	0·42	37/8897
278	*DNMT3A*	chr2:g.25463181	c.2312G>C	p.Arg771Pro	exact	0·41	32/7718
279	*DNMT3A*	chr2:g.25463181	c.2312G>A	p.Arg771Gln	exact	0·24	20/8328
280	*DNMT3A*	chr2:g.25463269	c.2224C>G	p.Arg742Gly	overlapping	3·60	147/4089
280	*DNMT3A*	chr2:g.25463182	c.2311C>T	p.Arg771Ter	exact	0·15	12/8058
281	*DNMT3A*	chr2:g.25463182	c.2311C>T	p.Arg771Ter	exact	0·27	15/5563
282	*DNMT3A*	chr2:g.25463182	c.2311C>T	p.Arg771Ter	exact	0·20	18/8990
283	*DNMT3A*	chr2:g.25463289	c.2204A>G	p.Tyr735Cys	exact	0·68	19/2807
283	*DNMT3A*	chr2:g.25463184	c.2309C>G	p.Ser770Trp	exact	0·32	27/8491
284	*DNMT3A*	chr2:g.25463184	c.2309C>A	p.Ser770Ter	exact	2·00	196/9800

285	*DNMT3A*	chr2:g.25463184	c.2309C>T	p.Ser770Leu	exact	0·24	23/9666
286	*DNMT3A*	chr2:g.25463184	c.2309C>T	p.Ser770Leu	exact	0·23	16/6902
287	*DNMT3A*	chr2:g.25463184	c.2309C>T	p.Ser770Leu	exact	0·64	61/9513
288	*DNMT3A*	chr2:g.25463184	c.2309C>T	p.Ser770Leu	exact	0·32	28/8797
289	*DNMT3A*	chr2:g.25463186	c.2307C>G	p.Ile769Met	overlapping	0·16	15/9550
290	*DNMT3A*	chr2:g.25463289	c.2204A>G	p.Tyr735Cys	exact	0·42	16/3830
290	*DNMT3A*	chr2:g.25463190	c.2303A>G	p.Asp768Gly	overlapping	0·11	11/9590
291	*DNMT3A*	chr2:g.25466800	c.1903C>G	p.Arg635Gly	exact	1·19	57/4781
291	*DNMT3A*	chr2:g.25463190	c.2303A>G	p.Asp768Gly	overlapping	0·02	2/9830
292	*DNMT3A*	chr2:g.25463190	c.2303A>G	p.Asp768Gly	overlapping	0·04	4/9826
293	*DNMT3A*	chr2:g.25463190	c.2303A>G	p.Asp768Gly	overlapping	0·01	1/9832
294	*DNMT3A*	chr2:g.25463190	c.2303A>G	p.Asp768Gly	overlapping	0·04	4/9798
295	*DNMT3A*	chr2:g.25463194	c.2299delA	p.Arg767Glyfs*12	new	4·07	160/3933
296	*DNMT3A*	chr2:g.25463229	c.2264T>C	p.Phe755Ser	exact	3·41	140/4101
297	*DNMT3A*	chr2:g.25463229	c.2264T>C	p.Phe755Ser	exact	0·41	17/4136
298	*DNMT3A*	chr2:g.25463230	c.2263T>A	p.Phe755Ile	exact	0·84	50/5957
299	*DNMT3A*	chr2:g.25463260	c.2233G>T	p.Glu745Ter	exact	1·79	85/4739
300	*DNMT3A*	chr2:g.25463272	c.2221G>C	p.Ala741Pro	overlapping	1·40	45/3217
300	*GNB1*	chr1:g.1747229	c.169A>G	p.Lys57Glu	exact	0·76	33/4365
301	*DNMT3A*	chr2:g.25463283	c.2210T>G	p.Leu737Arg	exact	1·15	50/4341
302	*DNMT3A*	chr2:g.25463286	c.2207G>A	p.Arg736His	exact	0·61	24/3918
303	*DNMT3A*	chr2:g.25463287	c.2206C>G	p.Arg736Gly	exact	0·52	19/3650
304	*DNMT3A*	chr2:g.25463287	c.2206C>T	p.Arg736Cys	exact	0·41	10/2449
305	*DNMT3A*	chr2:g.25463583	c.2099C>T	p.Pro700Leu	exact	0·38	19/5035
305	*DNMT3A*	chr2:g.25463289	c.2204A>G	p.Tyr735Cys	exact	7·51	315/4193
306	*DNMT3A*	chr2:g.25463289	c.2204A>G	p.Tyr735Cys	exact	17·70	686/3875
306	*DNMT3A*	chr2:g.25470923	c.838delG	p.Asp280Thrfs*36	new	1·36	35/2571
307	*DNMT3A*	chr2:g.25463289	c.2204A>G	p.Tyr735Cys	exact	0·68	21/3094
308	*DNMT3A*	chr2:g.25463289	c.2204A>G	p.Tyr735Cys	exact	0·41	22/5398
309	*DNMT3A*	chr2:g.25463289	c.2204A>G	p.Tyr735Cys	exact	0·50	11/2196
310	*DNMT3A*	chr2:g.25463289	c.2204A>G	p.Tyr735Cys	exact	1·11	36/3244

311	*DNMT3A*	chr2:g.25463289	c.2204A>G	p.Tyr735Cys	exact	0·24	9/3745
312	*DNMT3A*	chr2:g.25463289	c.2204A>C	p.Tyr735Ser	exact	2·83	110/3891
313	*DNMT3A*	chr2:g.25463289	c.2204A>G	p.Tyr735Cys	exact	0·32	13/4011
314	*DNMT3A*	chr2:g.25463289	c.2204A>G	p.Tyr735Cys	exact	0·78	27/3478
315	*DNMT3A*	chr2:g.25463289	c.2204A>G	p.Tyr735Cys	exact	0·19	8/4131
316	*DNMT3A*	chr2:g.25463289	c.2204A>G	p.Tyr735Cys	exact	0·21	8/3796
317	*DNMT3A*	chr2:g.25463289	c.2204A>G	p.Tyr735Cys	exact	3·20	84/2626
318	*DNMT3A*	chr2:g.25463296_ 25463297	c.2196_2197insAG	p.Glu733Argfs*47	exact	1·93	74/3825
319	*DNMT3A*	chr2:g.25463298	c.2195T>C	p.Phe732Ser	exact	2·52	110/4363
320	*DNMT3A*	chr2:g.25463298_ 25463300	c.2193_2195delCTT	p.Phe732del	exact	15·22	647/4250
320	*GNB1*	chr1:g.1747227	c.171G>T	p.Lys57Asn	exact	15·31	519/3391
321	*DNMT3A*	chr2:g.25463298_ 25463300	c.2193_2195delCTT	p.Phe732del	exact	1·89	46/2429
322	*DNMT3A*	chr2:g.25463298_ 25463300	c.2193_2195delCTT	p.Phe732del	exact	0·51	19/3727
323	*DNMT3A*	chr2:g.25463300	c.2193C>G	p.Phe731Leu	exact	0·63	45/7172
324	*DNMT3A*	chr2:g.25463307	c.2186G>A	p.Arg729Gln	exact	0·20	15/7667
325	*DNMT3A*	chr2:g.25469490	c.1278delA	p.Glu427Lysfs*224	exact	6·05	86/1421
325	*DNMT3A*	chr2:g.25463308	c.2185C>T	p.Arg729Trp	exact	0·51	24/4742
326	*DNMT3A*	chr2:g.25463308	c.2185C>T	p.Arg729Trp	exact	0·62	51/8260
327	*DNMT3A*	chr2:g.25463308	c.2185C>T	p.Arg729Trp	exact	0·69	35/5047
328	*DNMT3A*	chr2:g.25463308	c.2185C>T	p.Arg729Trp	exact	0·51	19/3712
329	*DNMT3A*	chr2:g.25463308	c.2185C>T	p.Arg729Trp	exact	1·12	94/8390
330	*DNMT3A*	chr2:g.25463316	c.2177delG	p.Gly726Alafs*53	exact	0·94	44/4673
331	*DNMT3A*	chr2:g.25463539_ 25463540	c.2142dupC	p.Ile715Hisfs*19	exact	6·69	114/1703
332	*DNMT3A*	chr2:g.25463541	c.2141C>T	p.Ser714Phe	overlapping	14·84	236/1590
333	*DNMT3A*	chr2:g.25463577	c.2105A>G	p.Asp702Gly	overlapping	0·65	21/3229
334	*DNMT3A*	chr2:g.25463583	c.2099C>T	p.Pro700Leu	exact	9·54	244/2557
335	*DNMT3A*	chr2:g.25463584	c.2098C>A	p.Pro700Thr	exact	0·64	14/2176
336	*DNMT3A*	chr2:g.25463586	c.2096G>A	p.Gly699Asp	exact	0·93	32/3444

337	*DNMT3A*	chr2:g.25463586	c.2096G>A	p.Gly699Asp	exact	2·44	40/1642
338	*DNMT3A*	chr2:g.25468150	c.1526delT	p.Phe509Serfs*142	exact	2·11	35/1660
338	*DNMT3A*	chr2:g.25463596	c.2086delC	p.Gln696Argfs*9	exact	2·82	85/3014
339	*DNMT3A*	chr2:g.25463596	c.2086C>T	p.Gln696Ter	exact	1·69	53/3139
340	*DNMT3A*	chr2:g.25464534	c.1979A>G	p.Tyr660Cys	exact	0·58	48/8338
341	*DNMT3A*	chr2:g.25464537	c.1976G>A	p.Arg659His	exact	0·13	9/6921
342	*DNMT3A*	chr2:g.25464541	c.1972G>T	p.Asp658Tyr	overlapping	1·59	46/2886
343	*DNMT3A*	chr2:g.25464544	c.1969G>A	p.Val657Met	exact	8·95	429/4795
344	*DNMT3A*	chr2:g.25464544	c.1969G>A	p.Val657Met	exact	0·73	16/2198
345	*DNMT3A*	chr2:g.25464570	c.1943T>C	p.Leu648Pro	new	0·20	11/5560
346	*DNMT3A*	chr2:g.25464570	c.1943T>C	p.Leu648Pro	new	5·56	169/3042
347	*DNMT3A*	chr2:g.25464570	c.1943T>C	p.Leu648Pro	new	0·20	10/5102
348	*DNMT3A*	chr2:g.25464576	c.1937G>A	p.Gly646Glu	exact	1·09	44/4044
349	*DNMT3A*	chr2:g.25466767	c.1936G>A	p.Gly646Arg	exact	0·71	14/1983
350	*DNMT3A*	chr2:g.25466797	c.1906G>A	p.Val636Met	exact	0·71	15/2114
351	*DNMT3A*	chr2:g.25466797	c.1906G>A	p.Val636Met	exact	20·93	736/3517
352	*DNMT3A*	chr2:g.25466799	c.1904G>T	p.Arg635Leu	exact	4·87	164/3370
353	*DNMT3A*	chr2:g.25466799	c.1904G>A	p.Arg635Gln	exact	5·17	132/2555
354	*DNMT3A*	chr2:g.25466800	c.1903C>T	p.Arg635Trp	exact	0·48	15/3155
355	*DNMT3A*	chr2:g.25467038	c.1837delC	p.His613Thrfs*38	exact	6·17	84/1362
356	*DNMT3A*	chr2:g.25467073	c.1802G>A	p.Trp601Ter	exact	5·57	92/1652
357	*DNMT3A*	chr2:g.25467083	c.1792C>T	p.Arg598Ter	exact	1·11	17/1536
358	*DNMT3A*	chr2:g.25467083	c.1792C>T	p.Arg598Ter	exact	6·51	134/2059
359	*DNMT3A*	chr2:g.25467087	c.1788delG	p.Arg597Glyfs*54	overlapping	2·74	82/2990
360	*DNMT3A*	chr2:g.25467091	c.1784T>C	p.Leu595Pro	exact	0·52	20/3867
360	*JAK2*	chr9:g.5073770	c.1849G>T	p.Val617Phe	exact	0·46	38/8186
361	*DNMT3A*	chr2:g.25467200	c.1675delT	p.Cys559Alafs*92	exact	10·21	69/676
362	*DNMT3A*	chr2:g.25467447_ 25467448	c.1628dupG	p.Arg544Profs*2	new	2·88	65/2259
363	*DNMT3A*	chr2:g.25467448	c.1628G>C	p.Gly543Ala	exact	4·79	64/1336
364	*DNMT3A*	chr2:g.25467449	c.1627G>T	p.Gly543Cys	exact	2·64	60/2276
365	*DNMT3A*	chr2:g.25468125	c.1551C>G	p.Cys517Trp	exact	1·49	51/3425

366	*DNMT3A*	chr2:g.25470480	c.994G>A	p.Gly332Arg	exact	0·31	15/4812
367	*DNMT3A*	chr2:g.25470484	c.990G>T	p.Trp330Cys	overlapping	0·68	38/5626
368	*DNMT3A*	chr2:g.25470485	c.989G>A	p.Trp330Ter	exact	0·74	28/3774
369	*DNMT3A*	chr2:g.25470516	c.958C>T	p.Arg320Ter	exact	0·29	13/4498
370	*DNMT3A*	chr2:g.25470516	c.958C>T	p.Arg320Ter	exact	0·68	42/6168
371	*DNMT3A*	chr2:g.25470516	c.958C>T	p.Arg320Ter	exact	3·54	107/3026
372	*DNMT3A*	chr2:g.25470533	c.941G>A	p.Trp314Ter	exact	12·72	43/338
373	*DNMT3A*	chr2:g.25470992_ 25470993	c.767_768dupCC	p.Thr257Profs*60	exact	4·14	87/2102
374	*GNAS*	chr20:g.57484414	c.2524C>T	p.Arg842Cys	new	0·09	9/9681
375	*GNAS*	chr20:g.57484414	c.2524C>T	p.Arg842Cys	new	0·13	10/7882
376	*GNAS*	chr20:g.57484417	c.2527T>C	p.Cys843Arg	new	0·14	12/8533
377	*GNAS*	chr20:g.57484420	c.2530C>T	p.Arg844Cys	exact	0·08	8/9681
377	*IDH2*	chr15:g.90631935	c.418C>T	p.Arg140Trp	exact	0·11	10/9285
378	*GNAS*	chr20:g.57484420	c.2530C>T	p.Arg844Cys	exact	1·19	114/9600
379	*GNAS*	chr20:g.57484421	c.2531G>A	p.Arg844His	exact	0·36	12/3375
380	*GNAS*	chr20:g.57484421	c.2531G>A	p.Arg844His	exact	0·21	19/9233
381	*GNAS*	chr20:g.57484421	c.2531G>A	p.Arg844His	exact	0·74	45/6070
382	*GNAS*	chr20:g.57484421	c.2531G>A	p.Arg844His	exact	0·42	32/7677
383	*GNB1*	chr1:g.1747229	c.169A>G	p.Lys57Glu	exact	1·25	84/6745
384	*GNB1*	chr1:g.1747229	c.169A>G	p.Lys57Glu	exact	1·17	48/4104
385	*GNB1*	chr1:g.1747229	c.169A>G	p.Lys57Glu	exact	0·19	17/8969
386	*GNB1*	chr1:g.1747229	c.169A>G	p.Lys57Glu	exact	0·24	17/7149
387	*GNB1*	chr1:g.1747229	c.169A>G	p.Lys57Glu	exact	0·30	16/5246
388	*IDH2*	chr15:g.90631934	c.419G>A	p.Arg140Gln	exact	9·52	268/2816
389	*IDH2*	chr15:g.90631934	c.419G>A	p.Arg140Gln	exact	1·07	46/4309
390	*IDH2*	chr15:g.90631935	c.418C>T	p.Arg140Trp	exact	0·09	8/9295
391	*JAK2*	chr9:g.5073770	c.1849G>T	p.Val617Phe	exact	7·06	189/2677
392	*JAK2*	chr9:g.5073770	c.1849G>T	p.Val617Phe	exact	5·39	258/4789
393	*KRAS*	chr12:g.25398281	c.38G>A	p.Gly13Asp	exact	0·89	43/4818
394	*KRAS*	chr12:g.25398285	c.34G>T	p.Gly12Cys	exact	1·21	63/5190
395	*SF3B1*	chr2:g.198266834	c.2098A>G	p.Lys700Glu	exact	0·31	28/8938

396	*SF3B1*	chr2:g.198267360	c.1997A>G	p.Lys666Arg	exact	6·80	82/1205
397	*SRSF2*	chr17:g.74732960	c.283C>G	p.Pro95Ala	exact	11·57	172/1487
398	*STAT3*	chr17:g.40474420	c.1981G>T	p.Asp661Tyr	exact	0·57	15/2651
399	*STAT3*	chr17:g.40474420	c.1981G>T	p.Asp661Tyr	exact	1·80	41/2280
400	*TET2*	chr4:g.106156729	c.1693C>T	p.Arg565Ter	new LoF *TET2*	0·41	27/6511
401	*TET2*	chr4:g.106156729	c.1693C>T	p.Arg565Ter	new LoF *TET2*	0·02	2/9592
402	*TET2*	chr4:g.106156747	c.1711C>T	p.Arg571Ter	exact	0·76	41/5378
403	*TET2*	chr4:g.106157725	c.2689C>T	p.Gln897Ter	overlapping	0·65	24/3671
404	*TP53*	chr17:g.7578410	c.520A>G	p.Arg174Gly	new	0·13	11/8349
405	*TP53*	chr17:g.7578410	c.520A>G	p.Arg174Gly	new	0·22	13/5785

a) *CHDMs in the literature are listed in Table S4a*
b) *We cannot exclude that some detected somatic mutations with very low VAF are false positives*
c) *Average variant and total read counts for PCR1 and PCR2. Generated by mpileups of all non-error-corrected reads; insertions and deletions are manually checked for read counts in the IGV browser*

Table S4a: CHDMs in literature – studies used for comparison in Tables S4b-d

**Study (first author, publication year)**	**PMID**
Bick, 2020	33057201
Busque, 2020	32492156
Desai, 2020	29988143
Genovese, 2014	25426838
Jaiswal, 2014	25426837
Jaiswal, 2017	28636844
McKerrel, 2015	25732814
Xie, 2014	25326804
Young, 2016	27546487
Zink, 2017	28483762

Table S4b: Overlap of CHDMs in the single-timepoint usual care group and CHDMs in literature as listed in Table S4a

	**total CHDM n (different CHDM n)**	**total CHDM % (different CHDM %)**	**VAF mean**
Known mutations in literature	218 (106)	79·85% (78·52%)	3·08
..of which reported ≥5 times	*169 (64)*	*61·90% (47·41%)*	*2·75*
New LoF in *DNMT3A, TET2, ASXL1*	8 (7)	2·93% (5·19%)	3·08
Known AA residue, new mutation	28 (17)	10·26% (12·59%)	1·32
New AA residue and mutation	19 (5)	6·96% (3·70%)	0·53
Sum	273 (135)		

Table S4c: Overlap of CHDMs in the single-timepoint bariatric surgery group and CHDMs in literature as listed in Table S4a

	**total CHDM n (different CHDM n)**	**total CHDM % (different CHDM %)**	**VAF mean**
Known mutations in literature	178 (94)	80·90% (75·81%)	2·52
..of which reported ≥5 times	*139 (51)*	*63·18% (41·13%)*	*2·27*
New LoF in *DNMT3A, TET2, ASXL1*	2 (1)	0·91% (0·81%)	0·22
Known AA residue, new mutation	25 (20)	11·36% (16·13%)	2·25
New AA residue and mutation	15 (9)	6·82% (7·26%)	2·06
Sum	220 (124)		

Table S4d: Overlap of CHDMs in the multiple-timepoint usual care group and CHDMs in the literature as listed in Table S4a

	**total CHDM n (different CHDM n)**	**total CHDM % (different CHDM %)**	**Events n**	**Growing trajectories n**	**Shrinking trajectories n**	**Static trajectories n**
Known mutations in literature	62 (34)	53·91% (64·15%)	7	25	5	25
..of which reported ≥5 times	*49 (25)*	*42·61% (47·17%)*	*5*	*21*	*3*	*20*
New LoF in *DNMT3A, TET2, ASXL1*	9 (5)	7·83% (9·43%)	5	2	0	2
Known AA residue, new mutation	19 (7)	16·52% (13·21%)	14	3	0	2
New AA residue and mutation	25 (7)	21·74% (13·21%)	12	0	0	13
Sum	115 (53)		38	30	5	42

Table S5: CHDMs identified in the multiple-timepoint usual care group

ID	Gene name	Location	HGVS c	HGVS p	Literature[a]	VAF[b]	Type of CHDM	Timepoint	Read count[c] variant / total
406	*ASXL1*	chr20:g.31022599_31022600	c.2083_2084dupCA	p.Gln695Hisfs*9	exact	0·63	static trajectory	A	61/9668
406	*ASXL1*	chr20:g.31022599_31022600	c.2083_2084dupCA	p.Gln695Hisfs*9	exact	0·78	static trajectory	B	28/3585
406	*ASXL1*	chr20:g.31022599_31022600	c.2083_2084dupCA	p.Gln695Hisfs*9	exact	0·15	static trajectory	D	12/7745
406	*ASXL1*	chr20:g.31022599_31022600	c.2083_2084dupCA	p.Gln695Hisfs*9	exact	0·38	static trajectory	E	6/1591
406	*DNMT3A*	chr2:g.25457243	c.2644C>T	p.Arg882Cys	exact	7·53	growing trajectory	A	733/9739
406	*DNMT3A*	chr2:g.25457243	c.2644C>T	p.Arg882Cys	exact	7·25	growing trajectory	B	202/2788
406	*DNMT3A*	chr2:g.25457243	c.2644C>T	p.Arg882Cys	exact	6·03	growing trajectory	C	317/5261
406	*DNMT3A*	chr2:g.25457243	c.2644C>T	p.Arg882Cys	exact	6·66	growing trajectory	D	639/9598
406	*DNMT3A*	chr2:g.25457243	c.2644C>T	p.Arg882Cys	exact	8·09	growing trajectory	E	531/6561
406	*DNMT3A*	chr2:g.25459831	c.2452T>C	p.Cys818Arg	overlapping	0·44	static trajectory	C	11/2482
406	*DNMT3A*	chr2:g.25459831	c.2452T>C	p.Cys818Arg	overlapping	0·18	static trajectory	D	17/9441
406	*DNMT3A*	chr2:g.25459831	c.2452T>C	p.Cys818Arg	overlapping	0·46	static trajectory	E	14/3019
406	*DNMT3A*	chr2:g.25469996	c.1046C>A	p.Ser349Ter	new LoF *DNMT3A*	0·74	growing trajectory	A	35/4758
406	*DNMT3A*	chr2:g.25469996	c.1046C>A	p.Ser349Ter	new LoF *DNMT3A*	1·58	growing trajectory	B	15/949
406	*DNMT3A*	chr2:g.25469996	c.1046C>A	p.Ser349Ter	new LoF *DNMT3A*	0·91	growing trajectory	C	21/2302
406	*DNMT3A*	chr2:g.25469996	c.1046C>A	p.Ser349Ter	new LoF *DNMT3A*	0·8	growing trajectory	D	45/5629
406	*DNMT3A*	chr2:g.25469996	c.1046C>A	p.Ser349Ter	new LoF *DNMT3A*	1·57	growing trajectory	E	48/3056
406	*TET2*	chr4:g.106156747	c.1711C>T	p.Arg571Ter	exact	0·35	static trajectory	A	34/9697
406	*TET2*	chr4:g.106156747	c.1711C>T	p.Arg571Ter	exact	0·19	static trajectory	B	9/4640
406	*TET2*	chr4:g.106156747	c.1711C>T	p.Arg571Ter	exact	0·14	static trajectory	C	4/2792
406	*TET2*	chr4:g.106156747	c.1711C>T	p.Arg571Ter	exact	0·16	static trajectory	D	15/9572

407	*BRCC3*	chrX:g.154305512	c.263A>G	p.Asp88Gly	new	0·92	event	C	11/1194
407	*DNMT3A*	chr2:g.25463308	c.2185C>T	p.Arg729Trp	exact	0·23	event	C	4/1709
407	*DNMT3A*	chr2:g.25463308	c.2185C>T	p.Arg729Trp	exact	0·52	event	D	40/7645
407	*GNAS*	chr20:g.57484417	c.2527T>C	p.Cys843Arg	new	0·09	static trajectory	A	9/9833
407	*GNAS*	chr20:g.57484417	c.2527T>C	p.Cys843Arg	new	0·77	static trajectory	C	16/2087
407	*GNAS*	chr20:g.57484417	c.2527T>C	p.Cys843Arg	new	0·08	static trajectory	D	8/9796
407	*NRAS*	chr1:g.115258721	c.61A>G	p.Ile21Val	new	1·19	event	C	13/1091
408	*BRCC3*	chrX:g.154305512	c.263A>G	p.Asp88Gly	new	0·69	static trajectory	A	9/1309
408	*BRCC3*	chrX:g.154305512	c.263A>G	p.Asp88Gly	new	0·44	static trajectory	B	5/1146
408	*BRCC3*	chrX:g.154305512	c.263A>G	p.Asp88Gly	new	0·38	static trajectory	C	8/2129
408	*DNMT3A*	chr2:g.25457243	c.2644C>T	p.Arg882Cys	exact	0·64	static trajectory	A	9/1416
408	*DNMT3A*	chr2:g.25457243	c.2644C>T	p.Arg882Cys	exact	0·74	static trajectory	B	8/1082
408	*DNMT3A*	chr2:g.25457243	c.2644C>T	p.Arg882Cys	exact	0·32	static trajectory	C	10/3127
408	*DNMT3A*	chr2:g.25457243	c.2644C>T	p.Arg882Cys	exact	0·49	static trajectory	E	9/1836
408	*GNAS*	chr20:g.57484417	c.2527T>C	p.Cys843Arg	new	0·44	static trajectory	A	15/3387
408	*GNAS*	chr20:g.57484417	c.2527T>C	p.Cys843Arg	new	0·62	static trajectory	B	20/3213
408	*GNAS*	chr20:g.57484417	c.2527T>C	p.Cys843Arg	new	0·64	static trajectory	C	18/2800
408	*GNAS*	chr20:g.57484417	c.2527T>C	p.Cys843Arg	new	0·32	static trajectory	D	19/5934
408	*GNAS*	chr20:g.57484417	c.2527T>C	p.Cys843Arg	new	0·26	static trajectory	E	16/6130
408	*NRAS*	chr1:g.115258721	c.61A>G	p.Ile21Val	new	0·93	static trajectory	A	15/1619
408	*NRAS*	chr1:g.115258721	c.61A>G	p.Ile21Val	new	1	static trajectory	B	13/1297
408	*NRAS*	chr1:g.115258721	c.61A>G	p.Ile21Val	new	0·56	static trajectory	C	13/2333
408	*NRAS*	chr1:g.115258721	c.61A>G	p.Ile21Val	new	0·31	static trajectory	D	7/2259
408	*NRAS*	chr1:g.115258721	c.61A>G	p.Ile21Val	new	0·44	static trajectory	E	12/2729
408	*TP53*	chr17:g.7578410	c.520A>G	p.Arg174Gly	new	0·58	event	A	6/1030
408	*TP53*	chr17:g.7578410	c.520A>G	p.Arg174Gly	new	1·09	event	B	8/733
409	*CBL*	chr11:g.119148974	c.1194C>A	p.His398Gln	overlapping	0·65	event	C	16/2445
409	*CBL*	chr11:g.119148974	c.1194C>A	p.His398Gln	overlapping	2·21	event	D	51/2309
409	*DNMT3A*	chr2:g.25470498	c.976C>T	p.Arg326Cys	exact	1·04	growing trajectory	A	15/1436
409	*DNMT3A*	chr2:g.25470498	c.976C>T	p.Arg326Cys	exact	1·78	growing trajectory	B	42/2362

409	*DNMT3A*	chr2:g.25470498	c.976C>T	p.Arg326Cys	exact	1·66	growing trajectory	C	42/2536
409	*DNMT3A*	chr2:g.25470498	c.976C>T	p.Arg326Cys	exact	1·75	growing trajectory	D	50/2860
410	*CBL*	chr11:g.119148991	c.1211G>A	p.Cys404Tyr	exact	3·21	growing trajectory	A	130/4049
410	*CBL*	chr11:g.119148991	c.1211G>A	p.Cys404Tyr	exact	4·37	growing trajectory	B	67/1534
410	*CBL*	chr11:g.119148991	c.1211G>A	p.Cys404Tyr	exact	28·94	growing trajectory	C	1145/3956
410	*CBL*	chr11:g.119148991	c.1211G>A	p.Cys404Tyr	exact	35·44	growing trajectory	D	735/2074
410	*CBL*	chr11:g.119148991	c.1211G>A	p.Cys404Tyr	exact	36·79	growing trajectory	E	2360/6415
410	*DNMT3A*	chr2:g.25458605_ 25458606	c.2567_2568delAG	p.Glu856Glyfs*7	exact	0·31	static trajectory	A	29/9463
410	*DNMT3A*	chr2:g.25458605_ 25458606	c.2567_2568delAG	p.Glu856Glyfs*7	exact	0·43	static trajectory	B	21/4907
410	*DNMT3A*	chr2:g.25458605_ 25458606	c.2567_2568delAG	p.Glu856Glyfs*7	exact	0·77	static trajectory	C	65/8413
410	*DNMT3A*	chr2:g.25458605_ 25458606	c.2567_2568delAG	p.Glu856Glyfs*7	exact	0·25	static trajectory	D	10/4066
410	*DNMT3A*	chr2:g.25458605_ 25458606	c.2567_2568delAG	p.Glu856Glyfs*7	exact	0·1	static trajectory	E	7/6794
410	*DNMT3A*	chr2:g.25459831	c.2452T>C	p.Cys818Arg	overlapping	0·21	event	E	16/7579
411	*CBL*	chr11:g.119149250	c.1258C>T	p.Arg420Ter	overlapping	0·05	event	C	4/7855
411	*CBL*	chr11:g.119149250	c.1258C>T	p.Arg420Ter	overlapping	0·06	event	D	6/9775
411	*DNMT3A*	chr2:g.25470533	c.941G>A	p.Trp314Ter	exact	4·48	event	E	31/692
412	*CBL*	chr11:g.119149251	c.1259G>A	p.Arg420Gln	exact	25·29	growing trajectory	A	1001/3958
412	*CBL*	chr11:g.119149251	c.1259G>A	p.Arg420Gln	exact	26·2	growing trajectory	B	1426/5443
412	*CBL*	chr11:g.119149251	c.1259G>A	p.Arg420Gln	exact	27·98	growing trajectory	C	479/1712
412	*CBL*	chr11:g.119149251	c.1259G>A	p.Arg420Gln	exact	28·01	growing trajectory	D	2126/7591
412	*DNMT3A*	chr2:g.25459831	c.2452T>C	p.Cys818Arg	overlapping	0·36	event	C	7/1959
412	*DNMT3A*	chr2:g.25459831	c.2452T>C	p.Cys818Arg	overlapping	0·25	event	D	16/6463

413	*DNMT3A*	chr2:g.25457171	c.2716A>T	p.Lys906Ter	new LoF *DNMT3A*	0·11	static trajectory	C	7/6646
413	*DNMT3A*	chr2:g.25457171	c.2716A>T	p.Lys906Ter	new LoF *DNMT3A*	0·23	static trajectory	D	15/6388
413	*DNMT3A*	chr2:g.25457171	c.2716A>T	p.Lys906Ter	new LoF *DNMT3A*	0·18	static trajectory	E	18/9776
413	*DNMT3A*	chr2:g.25457242	c.2645G>A	p.Arg882His	exact	0·08	growing trajectory	A	6/7935
413	*DNMT3A*	chr2:g.25457242	c.2645G>A	p.Arg882His	exact	0·09	growing trajectory	B	4/4545
413	*DNMT3A*	chr2:g.25457242	c.2645G>A	p.Arg882His	exact	0·89	growing trajectory	C	50/5608
413	*DNMT3A*	chr2:g.25457242	c.2645G>A	p.Arg882His	exact	1·86	growing trajectory	D	120/6469
413	*DNMT3A*	chr2:g.25457242	c.2645G>A	p.Arg882His	exact	2·72	growing trajectory	E	252/9268
413	*DNMT3A*	chr2:g.25463286	c.2207G>A	p.Arg736His	exact	0·26	static trajectory	A	10/3776
413	*DNMT3A*	chr2:g.25463286	c.2207G>A	p.Arg736His	exact	1·22	static trajectory	C	37/3023
413	*DNMT3A*	chr2:g.25463286	c.2207G>A	p.Arg736His	exact	0·84	static trajectory	D	28/3336
413	*DNMT3A*	chr2:g.25463286	c.2207G>A	p.Arg736His	exact	0·59	static trajectory	E	31/5250
413	*SF3B1*	chr2:g.198266827	c.2105G>A	p.Arg702Gln	new	0·08	event	B	8/9682
413	*SF3B1*	chr2:g.198266827	c.2105G>A	p.Arg702Gln	new	0·07	event	C	6/8378
413	*TET2*	chr4:g.106157714	c.2678T>C	p.Val893Ala	new	0·29	event	A	4/1389
413	*TET2*	chr4:g.106157714	c.2678T>C	p.Val893Ala	new	1·01	event	E	14/1388
414	*DNMT3A*	chr2:g.25457176	c.2711C>T	p.Pro904Leu	exact	0·12	static trajectory	A	11/8821
414	*DNMT3A*	chr2:g.25457176	c.2711C>T	p.Pro904Leu	exact	0·11	static trajectory	B	4/3707
414	*DNMT3A*	chr2:g.25457176	c.2711C>T	p.Pro904Leu	exact	0·07	static trajectory	D	7/9748
414	*DNMT3A*	chr2:g.25457176	c.2711C>T	p.Pro904Leu	exact	0·07	static trajectory	E	7/9709
414	*IDH2*	chr15:g.90631934	c.419G>A	p.Arg140Gln	exact	0·07	event	D	7/9696
414	*IDH2*	chr15:g.90631934	c.419G>A	p.Arg140Gln	exact	0·12	event	E	12/9716
414	*SF3B1*	chr2:g.198266827	c.2105G>A	p.Arg702Gln	new	0·06	event	A	6/9741
414	*TET2*	chr4:g.106156729	c.1693C>T	p.Arg565Ter	new LoF *TET2*	0·07	event	C	7/9626
414	*TET2*	chr4:g.106156729	c.1693C>T	p.Arg565Ter	new LoF *TET2*	0·06	event	D	6/9688
414	*TET2*	chr4:g.106157714	c.2678T>C	p.Val893Ala	new	0·23	static trajectory	A	5/2132
414	*TET2*	chr4:g.106157714	c.2678T>C	p.Val893Ala	new	0·28	static trajectory	C	13/4603

414	*TET2*	chr4:g.106157714	c.2678T>C	p.Val893Ala	new	0·34	static trajectory	D	14/4097
415	*DNMT3A*	chr2:g.25457176	c.2711C>T	p.Pro904Leu	exact	0·05	static trajectory	B	4/7626
415	*DNMT3A*	chr2:g.25457176	c.2711C>T	p.Pro904Leu	exact	0·11	static trajectory	C	7/6329
415	*DNMT3A*	chr2:g.25457176	c.2711C>T	p.Pro904Leu	exact	0·11	static trajectory	D	11/9586
415	*DNMT3A*	chr2:g.25457243	c.2644C>T	p.Arg882Cys	exact	0·15	static trajectory	B	8/5419
415	*DNMT3A*	chr2:g.25457243	c.2644C>T	p.Arg882Cys	exact	0·12	static trajectory	C	5/4214
415	*DNMT3A*	chr2:g.25457243	c.2644C>T	p.Arg882Cys	exact	0·2	static trajectory	D	19/9385
415	*IDH2*	chr15:g.90631934	c.419G>A	p.Arg140Gln	exact	0·05	event	A	4/8466
415	*IDH2*	chr15:g.90631934	c.419G>A	p.Arg140Gln	exact	0·1	event	B	10/9704
415	*TP53*	chr17:g.7578178	c.671A>T	p.Glu224Val	overlapping	0·07	event	D	7/9454
416	*DNMT3A*	chr2:g.25457242	c.2645G>A	p.Arg882His	exact	0·16	growing trajectory	A	11/7054
416	*DNMT3A*	chr2:g.25457242	c.2645G>A	p.Arg882His	exact	0·19	growing trajectory	B	4/2144
416	*DNMT3A*	chr2:g.25457242	c.2645G>A	p.Arg882His	exact	0·58	growing trajectory	C	56/9657
416	*DNMT3A*	chr2:g.25457242	c.2645G>A	p.Arg882His	exact	0·64	growing trajectory	D	20/3117
416	*DNMT3A*	chr2:g.25457242	c.2645G>A	p.Arg882His	exact	0·8	growing trajectory	E	34/4262
416	*DNMT3A*	chr2:g.25459831	c.2452T>C	p.Cys818Arg	overlapping	0·46	event	C	31/6754
416	*DNMT3A*	chr2:g.25459831	c.2452T>C	p.Cys818Arg	overlapping	0·44	event	D	8/1803
416	*DNMT3A*	chr2:g.25463184	c.2309C>T	p.Ser770Leu	exact	2·03	growing trajectory	A	199/9808
416	*DNMT3A*	chr2:g.25463184	c.2309C>T	p.Ser770Leu	exact	2·33	growing trajectory	B	86/3686
416	*DNMT3A*	chr2:g.25463184	c.2309C>T	p.Ser770Leu	exact	2·87	growing trajectory	C	280/9749
416	*DNMT3A*	chr2:g.25463184	c.2309C>T	p.Ser770Leu	exact	3·08	growing trajectory	D	144/4682
416	*DNMT3A*	chr2:g.25463184	c.2309C>T	p.Ser770Leu	exact	2·82	growing trajectory	E	175/6198
416	*DNMT3A*	chr2:g.25463286	c.2207G>A	p.Arg736His	exact	0·59	static trajectory	A	21/3544
416	*DNMT3A*	chr2:g.25463286	c.2207G>A	p.Arg736His	exact	0·89	static trajectory	C	64/7188
416	*DNMT3A*	chr2:g.25463286	c.2207G>A	p.Arg736His	exact	0·89	static trajectory	D	16/1798
416	*DNMT3A*	chr2:g.25463286	c.2207G>A	p.Arg736His	exact	0·71	static trajectory	E	16/2268

416	*DNMT3A*	chr2:g.25469525	c.1243C>T	p.Gln415Ter	exact	1·77	growing trajectory	A	29/1636
416	*DNMT3A*	chr2:g.25469525	c.1243C>T	p.Gln415Ter	exact	1·63	growing trajectory	B	9/551
416	*DNMT3A*	chr2:g.25469525	c.1243C>T	p.Gln415Ter	exact	4·09	growing trajectory	C	158/3860
416	*DNMT3A*	chr2:g.25469525	c.1243C>T	p.Gln415Ter	exact	4·25	growing trajectory	D	37/870
416	*DNMT3A*	chr2:g.25469525	c.1243C>T	p.Gln415Ter	exact	6·03	growing trajectory	E	61/1011
416	*TET2*	chr4:g.106156729	c.1693C>T	p.Arg565Ter	new LoF *TET2*	0·06	event	A	6/9761
416	*TET2*	chr4:g.106156729	c.1693C>T	p.Arg565Ter	new LoF *TET2*	0·09	event	D	5/5424
417	*DNMT3A*	chr2:g.25457242	c.2645G>A	p.Arg882His	exact	0·54	static trajectory	B	15/2763
417	*DNMT3A*	chr2:g.25457242	c.2645G>A	p.Arg882His	exact	0·78	static trajectory	C	22/2812
417	*DNMT3A*	chr2:g.25457242	c.2645G>A	p.Arg882His	exact	0·59	static trajectory	D	19/3213
418	*DNMT3A*	chr2:g.25457243	c.2644C>T	p.Arg882Cys	exact	0·16	static trajectory	A	14/8996
418	*DNMT3A*	chr2:g.25457243	c.2644C>T	p.Arg882Cys	exact	0·24	static trajectory	B	8/3346
418	*DNMT3A*	chr2:g.25457243	c.2644C>T	p.Arg882Cys	exact	0·31	static trajectory	C	16/5127
418	*DNMT3A*	chr2:g.25457243	c.2644C>T	p.Arg882Cys	exact	0·51	static trajectory	D	26/5067
418	*DNMT3A*	chr2:g.25457243	c.2644C>T	p.Arg882Cys	exact	0·54	static trajectory	E	31/5756
419	*DNMT3A*	chr2:g.25457243	c.2644C>T	p.Arg882Cys	exact	0·05	growing trajectory	B	3/5948
419	*DNMT3A*	chr2:g.25457243	c.2644C>T	p.Arg882Cys	exact	0·09	growing trajectory	C	5/5521
419	*DNMT3A*	chr2:g.25457243	c.2644C>T	p.Arg882Cys	exact	0·29	growing trajectory	D	28/9740
419	*DNMT3A*	chr2:g.25457243	c.2644C>T	p.Arg882Cys	exact	0·72	growing trajectory	E	9/1256
419	*DNMT3A*	chr2:g.25469614	c.1154delC	p.Pro385Argfs*22	exact	1·25	growing trajectory	B	10/798
419	*DNMT3A*	chr2:g.25469614	c.1154delC	p.Pro385Argfs*22	exact	2·26	growing trajectory	C	20/884
419	*DNMT3A*	chr2:g.25469614	c.1154delC	p.Pro385Argfs*22	exact	4·45	growing trajectory	D	61/1371
420	*DNMT3A*	chr2:g.25457243	c.2644C>T	p.Arg882Cys	exact	1·18	growing trajectory	A	29/2460
420	*DNMT3A*	chr2:g.25457243	c.2644C>T	p.Arg882Cys	exact	1·07	growing trajectory	B	35/3260

420	*DNMT3A*	chr2:g.25457243	c.2644C>T	p.Arg882Cys	exact	2·37	growing trajectory	C	88/3711
420	*DNMT3A*	chr2:g.25457243	c.2644C>T	p.Arg882Cys	exact	2·98	growing trajectory	D	77/2584
420	*GNAS*	chr20:g.57484417	c.2527T>C	p.Cys843Arg	new	0·13	static trajectory	A	7/5367
420	*GNAS*	chr20:g.57484417	c.2527T>C	p.Cys843Arg	new	0·05	static trajectory	B	5/9263
420	*GNAS*	chr20:g.57484417	c.2527T>C	p.Cys843Arg	new	0·53	static trajectory	D	15/2835
421	*DNMT3A*	chr2:g.25457249	c.2638A>G	p.Met880Val	exact	0·5	static trajectory	A	9/1817
421	*DNMT3A*	chr2:g.25457249	c.2638A>G	p.Met880Val	exact	0·4	static trajectory	B	14/3460
421	*DNMT3A*	chr2:g.25457249	c.2638A>G	p.Met880Val	exact	0·58	static trajectory	C	23/3985
421	*DNMT3A*	chr2:g.25457249	c.2638A>G	p.Met880Val	exact	0·61	static trajectory	D	59/9724
421	*GNAS*	chr20:g.57484417	c.2527T>C	p.Cys843Arg	new	0·63	static trajectory	A	27/4301
421	*GNAS*	chr20:g.57484417	c.2527T>C	p.Cys843Arg	new	0·16	static trajectory	B	11/6879
421	*GNAS*	chr20:g.57484417	c.2527T>C	p.Cys843Arg	new	0·31	static trajectory	C	18/5892
421	*NRAS*	chr1:g.115258721	c.61A>G	p.Ile21Val	new	0·72	static trajectory	A	15/2079
421	*NRAS*	chr1:g.115258721	c.61A>G	p.Ile21Val	new	0·34	static trajectory	B	15/4443
421	*NRAS*	chr1:g.115258721	c.61A>G	p.Ile21Val	new	0·26	static trajectory	C	11/4269
421	*TET2*	chr4:g.106156729	c.1693C>T	p.Arg565Ter	new LoF *TET2*	0·05	event	C	5/9729
421	*TET2*	chr4:g.106156729	c.1693C>T	p.Arg565Ter	new LoF *TET2*	0·09	event	D	9/9682
421	*TP53*	chr17:g.7578178	c.671A>T	p.Glu224Val	overlapping	0·09	event	D	8/9067
422	*DNMT3A*	chr2:g.25457249_25457252	c.2635_2638delAACA	p.Asn879Ter	new LoF *DNMT3A*	0·23	static trajectory	C	17/7424
422	*DNMT3A*	chr2:g.25457249_25457252	c.2635_2638delAACA	p.Asn879Ter	new LoF *DNMT3A*	0·3	static trajectory	D	25/8281
422	*DNMT3A*	chr2:g.25457249_25457252	c.2635_2638delAACA	p.Asn879Ter	new LoF *DNMT3A*	0·61	static trajectory	E	59/9686
422	*DNMT3A*	chr2:g.25459831	c.2452T>C	p.Cys818Arg	overlapping	0·08	static trajectory	C	5/6118
422	*DNMT3A*	chr2:g.25459831	c.2452T>C	p.Cys818Arg	overlapping	0·16	static trajectory	D	12/7631
422	*DNMT3A*	chr2:g.25459831	c.2452T>C	p.Cys818Arg	overlapping	0·1	static trajectory	E	9/8863
423	*DNMT3A*	chr2:g.25458684	c.2489T>C	p.Val830Ala	overlapping	0·72	growing trajectory	A	70/9680
423	*DNMT3A*	chr2:g.25458684	c.2489T>C	p.Val830Ala	overlapping	1·72	growing trajectory	B	162/9428
423	*DNMT3A*	chr2:g.25458684	c.2489T>C	p.Val830Ala	overlapping	3·04	growing trajectory	C	56/1841

423	*DNMT3A*	chr2:g.25458684	c.2489T>C	p.Val830Ala	overlapping	2·96	growing trajectory	D	45/1522
423	*DNMT3A*	chr2:g.25458684	c.2489T>C	p.Val830Ala	overlapping	2·44	growing trajectory	E	156/6382
423	*DNMT3A*	chr2:g.25459831	c.2452T>C	p.Cys818Arg	overlapping	0·53	event	E	16/2999
423	*DNMT3A*	chr2:g.25464534	c.1979A>G	p.Tyr660Cys	exact	0·08	growing trajectory	A	8/9815
423	*DNMT3A*	chr2:g.25464534	c.1979A>G	p.Tyr660Cys	exact	0·17	growing trajectory	B	12/7242
423	*DNMT3A*	chr2:g.25464534	c.1979A>G	p.Tyr660Cys	exact	0·4	growing trajectory	C	5/1254
423	*DNMT3A*	chr2:g.25464534	c.1979A>G	p.Tyr660Cys	exact	0·96	growing trajectory	D	11/1141
423	*DNMT3A*	chr2:g.25464534	c.1979A>G	p.Tyr660Cys	exact	1·29	growing trajectory	E	46/3554
423	*TET2*	chr4:g.106156747	c.1711C>T	p.Arg571Ter	exact	0·12	event	A	12/9685
423	*TET2*	chr4:g.106156747	c.1711C>T	p.Arg571Ter	exact	0·06	event	B	6/9536
424	*DNMT3A*	chr2:g.25459831	c.2452T>C	p.Cys818Arg	overlapping	0·18	event	D	18/9747
424	*DNMT3A*	chr2:g.25459831	c.2452T>C	p.Cys818Arg	overlapping	0·15	event	E	5/3260
424	*GNB1*	chr1:g.1747229	c.169A>G	p.Lys57Glu	exact	0·58	static trajectory	A	47/8165
424	*GNB1*	chr1:g.1747229	c.169A>G	p.Lys57Glu	exact	0·88	static trajectory	B	50/5714
424	*GNB1*	chr1:g.1747229	c.169A>G	p.Lys57Glu	exact	0·75	static trajectory	C	73/9690
424	*GNB1*	chr1:g.1747229	c.169A>G	p.Lys57Glu	exact	0·9	static trajectory	D	88/9760
424	*GNB1*	chr1:g.1747229	c.169A>G	p.Lys57Glu	exact	0·67	static trajectory	E	33/4899
425	*DNMT3A*	chr2:g.25459831	c.2452T>C	p.Cys818Arg	overlapping	0·37	event	D	11/2951
425	*DNMT3A*	chr2:g.25459831	c.2452T>C	p.Cys818Arg	overlapping	1·6	event	E	14/873
426	*DNMT3A*	chr2:g.25459831	c.2452T>C	p.Cys818Arg	overlapping	0·29	event	E	19/6580
426	*GNB1*	chr1:g.1747229	c.169A>G	p.Lys57Glu	exact	0·23	growing trajectory	A	15/6541
426	*GNB1*	chr1:g.1747229	c.169A>G	p.Lys57Glu	exact	0·19	growing trajectory	B	12/6258
426	*GNB1*	chr1:g.1747229	c.169A>G	p.Lys57Glu	exact	0·8	growing trajectory	C	54/6727
426	*GNB1*	chr1:g.1747229	c.169A>G	p.Lys57Glu	exact	1·17	growing trajectory	D	113/9653
426	*GNB1*	chr1:g.1747229	c.169A>G	p.Lys57Glu	exact	0·79	growing trajectory	E	76/9678

426	*TP53*	chr17:g.7578178	c.671A>T	p.Glu224Val	overlapping	0·06	event	E	4/7222
427	*DNMT3A*	chr2:g.25459831	c.2452T>C	p.Cys818Arg	overlapping	0·08	growing trajectory	A	4/4948
427	*DNMT3A*	chr2:g.25459831	c.2452T>C	p.Cys818Arg	overlapping	0·36	growing trajectory	C	16/4481
427	*DNMT3A*	chr2:g.25459831	c.2452T>C	p.Cys818Arg	overlapping	0·59	growing trajectory	D	25/4236
427	*GNAS*	chr20:g.57484417	c.2527T>C	p.Cys843Arg	new	0·14	static trajectory	A	4/4948
427	*GNAS*	chr20:g.57484417	c.2527T>C	p.Cys843Arg	new	0·12	static trajectory	B	16/4481
427	*GNAS*	chr20:g.57484417	c.2527T>C	p.Cys843Arg	new	0·14	static trajectory	D	25/4236
427	*GNAS*	chr20:g.57484421	c.2531G>A	p.Arg844His	exact	0·43	static trajectory	A	37/8606
427	*GNAS*	chr20:g.57484421	c.2531G>A	p.Arg844His	exact	0·43	static trajectory	B	22/5111
427	*GNAS*	chr20:g.57484421	c.2531G>A	p.Arg844His	exact	0·41	static trajectory	C	14/3431
427	*GNAS*	chr20:g.57484421	c.2531G>A	p.Arg844His	exact	0·14	static trajectory	D	4/2891
427	*GNAS*	chr20:g.57484421	c.2531G>A	p.Arg844His	exact	0·2	static trajectory	E	18/8971
428	*DNMT3A*	chr2:g.25462068	c.2339T>C	p.Ile780Thr	exact	0·33	static trajectory	C	16/4888
428	*DNMT3A*	chr2:g.25462068	c.2339T>C	p.Ile780Thr	exact	0·78	static trajectory	D	37/4750
428	*DNMT3A*	chr2:g.25462068	c.2339T>C	p.Ile780Thr	exact	0·75	static trajectory	E	23/3082
428	*IDH2*	chr15:g.90631934	c.419G>A	p.Arg140Gln	exact	0·69	static trajectory	A	66/9551
428	*IDH2*	chr15:g.90631934	c.419G>A	p.Arg140Gln	exact	0·95	static trajectory	B	53/5586
428	*IDH2*	chr15:g.90631934	c.419G>A	p.Arg140Gln	exact	0·9	static trajectory	C	87/9702
428	*IDH2*	chr15:g.90631934	c.419G>A	p.Arg140Gln	exact	0·87	static trajectory	D	85/9715
428	*IDH2*	chr15:g.90631934	c.419G>A	p.Arg140Gln	exact	0·45	static trajectory	E	40/8929
429	*DNMT3A*	chr2:g.25462068	c.2339T>C	p.Ile780Thr	exact	0·4	growing trajectory	A	10/2524
429	*DNMT3A*	chr2:g.25462068	c.2339T>C	p.Ile780Thr	exact	0·41	growing trajectory	B	13/3148
429	*DNMT3A*	chr2:g.25462068	c.2339T>C	p.Ile780Thr	exact	1·97	growing trajectory	C	54/2748
429	*DNMT3A*	chr2:g.25463190	c.2303A>G	p.Asp768Gly	overlapping	0·04	event	B	4/9599
429	*DNMT3A*	chr2:g.25463190	c.2303A>G	p.Asp768Gly	overlapping	0·11	event	C	8/7180
429	*DNMT3A*	chr2:g.25466790	c.1913C>T	p.Ser638Phe	exact	0·2	event	B	9/4551
429	*DNMT3A*	chr2:g.25466790	c.1913C>T	p.Ser638Phe	exact	0·71	event	C	19/2666
429	*GNAS*	chr20:g.57484417	c.2527T>C	p.Cys843Arg	new	0·16	event	D	16/9767

429	*TET2*	chr4:g.106156729	c.1693C>T	p.Arg565Ter	new LoF *TET2*	0·08	event	B	8/9753
430	*DNMT3A*	chr2:g.25463182	c.2311C>T	p.Arg771Ter	exact	0·65	shrinking trajectory	A	62/9570
430	*DNMT3A*	chr2:g.25463182	c.2311C>T	p.Arg771Ter	exact	0·32	shrinking trajectory	B	24/7424
430	*DNMT3A*	chr2:g.25463182	c.2311C>T	p.Arg771Ter	exact	0·38	shrinking trajectory	C	36/9419
430	*DNMT3A*	chr2:g.25463182	c.2311C>T	p.Arg771Ter	exact	0·09	shrinking trajectory	E	9/9786
430	*TET2*	chr4:g.106156747	c.1711C>T	p.Arg571Ter	exact	0·09	static trajectory	A	9/9726
430	*TET2*	chr4:g.106156747	c.1711C>T	p.Arg571Ter	exact	0·11	static trajectory	B	9/8238
430	*TET2*	chr4:g.106156747	c.1711C>T	p.Arg571Ter	exact	0·16	static trajectory	C	15/9575
430	*TET2*	chr4:g.106156747	c.1711C>T	p.Arg571Ter	exact	0·19	static trajectory	E	18/9713
431	*DNMT3A*	chr2:g.25463184	c.2309C>T	p.Ser770Leu	exact	0·16	static trajectory	A	11/6968
431	*DNMT3A*	chr2:g.25463184	c.2309C>T	p.Ser770Leu	exact	0·13	static trajectory	B	13/9757
431	*DNMT3A*	chr2:g.25463184	c.2309C>T	p.Ser770Leu	exact	0·23	static trajectory	C	14/5966
431	*DNMT3A*	chr2:g.25463184	c.2309C>T	p.Ser770Leu	exact	0·17	static trajectory	D	14/8074
431	*DNMT3A*	chr2:g.25463184	c.2309C>T	p.Ser770Leu	exact	0·11	static trajectory	E	11/9786
431	*DNMT3A*	chr2:g.25463298_25463300	c.2193_2195delCTT	p.Phe732del	exact	0·4	static trajectory	A	12/3017
431	*DNMT3A*	chr2:g.25463298_25463300	c.2193_2195delCTT	p.Phe732del	exact	0·27	static trajectory	B	12/4402
431	*DNMT3A*	chr2:g.25463298_25463300	c.2193_2195delCTT	p.Phe732del	exact	0·36	static trajectory	D	17/4690
431	*DNMT3A*	chr2:g.25463298_25463300	c.2193_2195delCTT	p.Phe732del	exact	0·13	static trajectory	E	7/5538
431	*DNMT3A*	chr2:g.25463299	c.2194T>A	p.Phe732Ile	exact	0·51	growing trajectory	A	20/3916
431	*DNMT3A*	chr2:g.25463299	c.2194T>A	p.Phe732Ile	exact	1·33	growing trajectory	B	81/6081
431	*DNMT3A*	chr2:g.25463299	c.2194T>A	p.Phe732Ile	exact	12·27	growing trajectory	C	535/4362
431	*DNMT3A*	chr2:g.25463299	c.2194T>A	p.Phe732Ile	exact	24·87	growing trajectory	D	1615/6495
431	*DNMT3A*	chr2:g.25463299	c.2194T>A	p.Phe732Ile	exact	35·02	growing trajectory	E	2808/8019
431	*TET2*	chr4:g.106157653	c.2617G>T	p.Glu873Ter	new LoF *TET2*	0·16	growing trajectory	A	9/5734

431	*TET2*	chr4:g.106157653	c.2617G>T	p.Glu873Ter	new LoF *TET2*	0·86	growing trajectory	B	56/6479
431	*TET2*	chr4:g.106157653	c.2617G>T	p.Glu873Ter	new LoF *TET2*	10·58	growing trajectory	C	470/4443
431	*TET2*	chr4:g.106157653	c.2617G>T	p.Glu873Ter	new LoF *TET2*	25·28	growing trajectory	D	1768/6994
431	*TET2*	chr4:g.106157653	c.2617G>T	p.Glu873Ter	new LoF *TET2*	37·1	growing trajectory	E	2569/6924
432	*DNMT3A*	chr2:g.25463190	c.2303A>G	p.Asp768Gly	overlapping	0·1	event	C	9/8710
432	*DNMT3A*	chr2:g.25463190	c.2303A>G	p.Asp768Gly	overlapping	0·06	event	E	6/9810
432	*DNMT3A*	chr2:g.25463576	c.2106T>G	p.Asp702Glu	exact	1·1	static trajectory	A	55/5019
432	*DNMT3A*	chr2:g.25463576	c.2106T>G	p.Asp702Glu	exact	1·47	static trajectory	B	33/2242
432	*DNMT3A*	chr2:g.25463576	c.2106T>G	p.Asp702Glu	exact	2·85	static trajectory	C	76/2663
432	*DNMT3A*	chr2:g.25463576	c.2106T>G	p.Asp702Glu	exact	1·11	static trajectory	E	54/4856
432	*DNMT3A*	chr2:g.25467173_25467175	c.1700_1702delTGG	p.Val567del	new	2·9	event	E	161/5554
432	*GNB1*	chr1:g.1747229	c.169A>G	p.Lys57Glu	exact	2·06	growing trajectory	A	199/9679
432	*GNB1*	chr1:g.1747229	c.169A>G	p.Lys57Glu	exact	1·76	growing trajectory	B	81/4600
432	*GNB1*	chr1:g.1747229	c.169A>G	p.Lys57Glu	exact	4·41	growing trajectory	C	191/4328
432	*GNB1*	chr1:g.1747229	c.169A>G	p.Lys57Glu	exact	21·98	growing trajectory	E	1522/6924
432	*TET2*	chr4:g.106156729	c.1693C>T	p.Arg565Ter	new LoF *TET2*	0·05	event	A	5/9772
433	*DNMT3A*	chr2:g.25463286	c.2207G>A	p.Arg736His	exact	0·46	static trajectory	C	17/3690
433	*DNMT3A*	chr2:g.25463286	c.2207G>A	p.Arg736His	exact	0·63	static trajectory	D	24/3790
433	*DNMT3A*	chr2:g.25463286	c.2207G>A	p.Arg736His	exact	0·93	static trajectory	E	56/5991
433	*DNMT3A*	chr2:g.25470516	c.958C>T	p.Arg320Ter	exact	0·39	static trajectory	C	13/3332
433	*DNMT3A*	chr2:g.25470516	c.958C>T	p.Arg320Ter	exact	0·53	static trajectory	D	16/3000
433	*DNMT3A*	chr2:g.25470516	c.958C>T	p.Arg320Ter	exact	0·74	static trajectory	E	38/5153
434	*DNMT3A*	chr2:g.25463287	c.2206C>G	p.Arg736Gly	exact	0·67	growing trajectory	A	14/2087
434	*DNMT3A*	chr2:g.25463287	c.2206C>G	p.Arg736Gly	exact	0·55	growing trajectory	B	8/1450
434	*DNMT3A*	chr2:g.25463287	c.2206C>G	p.Arg736Gly	exact	2·22	growing trajectory	C	86/3874

434	*DNMT3A*	chr2:g.25463287	c.2206C>G	p.Arg736Gly	exact	1·93	growing trajectory	D	50/2596
434	*DNMT3A*	chr2:g.25467083	c.1792C>T	p.Arg598Ter	exact	0·43	static trajectory	A	10/2340
434	*DNMT3A*	chr2:g.25467083	c.1792C>T	p.Arg598Ter	exact	0·53	static trajectory	B	11/2071
434	*DNMT3A*	chr2:g.25467083	c.1792C>T	p.Arg598Ter	exact	0·82	static trajectory	C	36/4379
434	*DNMT3A*	chr2:g.25467083	c.1792C>T	p.Arg598Ter	exact	0·62	static trajectory	D	12/1925
434	*JAK2*	chr9:g.5073770	c.1849G>T	p.Val617Phe	exact	0·15	growing trajectory	A	14/9239
434	*JAK2*	chr9:g.5073770	c.1849G>T	p.Val617Phe	exact	0·23	growing trajectory	B	9/3905
434	*JAK2*	chr9:g.5073770	c.1849G>T	p.Val617Phe	exact	0·23	growing trajectory	C	23/9829
434	*JAK2*	chr9:g.5073770	c.1849G>T	p.Val617Phe	exact	0·66	growing trajectory	D	36/5491
435	*DNMT3A*	chr2:g.25463289	c.2204A>G	p.Tyr735Cys	exact	0·3	static trajectory	A	13/4334
435	*DNMT3A*	chr2:g.25463289	c.2204A>G	p.Tyr735Cys	exact	0·55	static trajectory	D	3/545
435	*DNMT3A*	chr2:g.25463289	c.2204A>G	p.Tyr735Cys	exact	0·34	static trajectory	E	12/3524
436	*DNMT3A*	chr2:g.25463289	c.2204A>G	p.Tyr735Cys	exact	0·61	growing trajectory	A	19/3136
436	*DNMT3A*	chr2:g.25463289	c.2204A>G	p.Tyr735Cys	exact	0·81	growing trajectory	B	15/1857
436	*DNMT3A*	chr2:g.25463289	c.2204A>G	p.Tyr735Cys	exact	1·03	growing trajectory	C	39/3780
436	*DNMT3A*	chr2:g.25463289	c.2204A>G	p.Tyr735Cys	exact	1·03	growing trajectory	D	48/4660
436	*DNMT3A*	chr2:g.25463289	c.2204A>G	p.Tyr735Cys	exact	1·15	growing trajectory	E	77/6698
436	*TET2*	chr4:g.106157714	c.2678T>C	p.Val893Ala	new	0·33	event	D	9/2733
437	*DNMT3A*	chr2:g.25463289	c.2204A>G	p.Tyr735Cys	exact	0·27	event	C	6/2229
437	*DNMT3A*	chr2:g.25463289	c.2204A>G	p.Tyr735Cys	exact	0·32	event	D	19/5992
437	*DNMT3A*	chr2:g.25463308	c.2185C>T	p.Arg729Trp	exact	0·19	growing trajectory	B	6/3208
437	*DNMT3A*	chr2:g.25463308	c.2185C>T	p.Arg729Trp	exact	0·15	growing trajectory	C	6/4111
437	*DNMT3A*	chr2:g.25463308	c.2185C>T	p.Arg729Trp	exact	0·74	growing trajectory	D	72/9673
437	*GNAS*	chr20:g.57484417	c.2527T>C	p.Cys843Arg	new	0·08	static trajectory	A	5/6530

437	*GNAS*	chr20:g.57484417	c.2527T>C	p.Cys843Arg	new	0·41	static trajectory	B	26/6366
437	*GNAS*	chr20:g.57484417	c.2527T>C	p.Cys843Arg	new	0·39	static trajectory	C	24/6145
437	*IDH2*	chr15:g.90631934	c.419G>T	p.Arg140Leu	overlapping	0·16	growing trajectory	B	10/6325
437	*IDH2*	chr15:g.90631934	c.419G>T	p.Arg140Leu	overlapping	0·52	growing trajectory	C	31/6004
437	*IDH2*	chr15:g.90631934	c.419G>T	p.Arg140Leu	overlapping	0·68	growing trajectory	D	65/9590
437	*NRAS*	chr1:g.115258721	c.61A>G	p.Ile21Val	new	0·16	static trajectory	A	4/2579
437	*NRAS*	chr1:g.115258721	c.61A>G	p.Ile21Val	new	0·4	static trajectory	B	15/3731
437	*NRAS*	chr1:g.115258721	c.61A>G	p.Ile21Val	new	0·48	static trajectory	C	21/4408
437	*NRAS*	chr1:g.115258721	c.61A>G	p.Ile21Val	new	0·06	static trajectory	D	6/9657
438	*DNMT3A*	chr2:g.25463289	c.2204A>G	p.Tyr735Cys	exact	1·09	growing trajectory	A	19/1746
438	*DNMT3A*	chr2:g.25463289	c.2204A>G	p.Tyr735Cys	exact	1·21	growing trajectory	B	14/1160
438	*DNMT3A*	chr2:g.25463289	c.2204A>G	p.Tyr735Cys	exact	1·88	growing trajectory	C	33/1752
438	*DNMT3A*	chr2:g.25463289	c.2204A>G	p.Tyr735Cys	exact	1·95	growing trajectory	D	45/2309
439	*DNMT3A*	chr2:g.25463289	c.2204A>G	p.Tyr735Cys	exact	0·13	growing trajectory	A	7/5274
439	*DNMT3A*	chr2:g.25463289	c.2204A>G	p.Tyr735Cys	exact	0·5	growing trajectory	B	18/3624
439	*DNMT3A*	chr2:g.25463289	c.2204A>G	p.Tyr735Cys	exact	0·66	growing trajectory	C	9/1372
439	*DNMT3A*	chr2:g.25469167_25469168	c.1290dupT	p.Pro431Serfs*14	exact	0·32	growing trajectory	A	24/7489
439	*DNMT3A*	chr2:g.25469167_25469168	c.1290dupT	p.Pro431Serfs*14	exact	0·51	growing trajectory	B	26/5065
439	*DNMT3A*	chr2:g.25469167_25469168	c.1290dupT	p.Pro431Serfs*14	exact	3·42	growing trajectory	C	62/1812
439	*DNMT3A*	chr2:g.25469167_25469168	c.1290dupT	p.Pro431Serfs*14	exact	8·6	growing trajectory	D	181/2105
440	*DNMT3A*	chr2:g.25463308	c.2185C>T	p.Arg729Trp	exact	1·29	static trajectory	A	62/4799
440	*DNMT3A*	chr2:g.25463308	c.2185C>T	p.Arg729Trp	exact	1·28	static trajectory	C	78/6076
440	*DNMT3A*	chr2:g.25463308	c.2185C>T	p.Arg729Trp	exact	1	static trajectory	D	30/3013

440	*DNMT3A*	chr2:g.25466767	c.1936G>A	p.Gly646Arg	exact	2·33	shrinking trajectory	A	97/4157
440	*DNMT3A*	chr2:g.25466767	c.1936G>A	p.Gly646Arg	exact	2·59	shrinking trajectory	C	102/3935
440	*DNMT3A*	chr2:g.25466767	c.1936G>A	p.Gly646Arg	exact	0·97	shrinking trajectory	D	24/2475
440	*GNAS*	chr20:g.57484417	c.2527T>C	p.Cys843Arg	new	0·3	event	C	21/7028
440	*GNAS*	chr20:g.57484417	c.2527T>C	p.Cys843Arg	new	0·21	event	D	19/9258
440	*NRAS*	chr1:g.115258721	c.61A>G	p.Ile21Val	new	0·06	static trajectory	A	5/8150
440	*NRAS*	chr1:g.115258721	c.61A>G	p.Ile21Val	new	0·5	static trajectory	C	26/5177
440	*NRAS*	chr1:g.115258721	c.61A>G	p.Ile21Val	new	0·19	static trajectory	D	8/4324
440	*TP53*	chr17:g.7578410	c.520A>G	p.Arg174Gly	new	0·12	static trajectory	A	5/4244
440	*TP53*	chr17:g.7578410	c.520A>G	p.Arg174Gly	new	0·26	static trajectory	C	13/5006
440	*TP53*	chr17:g.7578410	c.520A>G	p.Arg174Gly	new	0·34	static trajectory	D	8/2387
441	*DNMT3A*	chr2:g.25463588	c.2094G>A	p.Trp698Ter	exact	11·41	shrinking trajectory	A	174/1525
441	*DNMT3A*	chr2:g.25463588	c.2094G>A	p.Trp698Ter	exact	10·79	shrinking trajectory	B	224/2076
441	*DNMT3A*	chr2:g.25463588	c.2094G>A	p.Trp698Ter	exact	7·77	shrinking trajectory	C	130/1674
441	*DNMT3A*	chr2:g.25463588	c.2094G>A	p.Trp698Ter	exact	6·11	shrinking trajectory	D	99/1619
442	*GNAS*	chr20:g.57484417	c.2527T>C	p.Cys843Arg	new	0·15	event	E	15/9762
442	*JAK2*	chr9:g.5073770	c.1849G>T	p.Val617Phe	exact	1·3	shrinking trajectory	A	128/9848
442	*JAK2*	chr9:g.5073770	c.1849G>T	p.Val617Phe	exact	0·9	shrinking trajectory	B	64/7117
442	*JAK2*	chr9:g.5073770	c.1849G>T	p.Val617Phe	exact	0·64	shrinking trajectory	C	5/787
442	*JAK2*	chr9:g.5073770	c.1849G>T	p.Val617Phe	exact	0·33	shrinking trajectory	D	11/3326
442	*JAK2*	chr9:g.5073770	c.1849G>T	p.Val617Phe	exact	0·11	shrinking trajectory	E	11/9650
442	*TP53*	chr17:g.7578410	c.520A>G	p.Arg174Gly	new	0·22	event	D	7/3217
443	*GNAS*	chr20:g.57484421	c.2531G>A	p.Arg844His	exact	0·83	growing trajectory	A	67/8079
443	*GNAS*	chr20:g.57484421	c.2531G>A	p.Arg844His	exact	0·98	growing trajectory	B	68/6932

443	*GNAS*	chr20:g.57484421	c.2531G>A	p.Arg844His	exact	1·61	growing trajectory	C	29/1802
443	*GNAS*	chr20:g.57484421	c.2531G>A	p.Arg844His	exact	3·56	growing trajectory	D	69/1937
444	*GNB1*	chr1:g.1747229	c.169A>G	p.Lys57Glu	exact	3·27	shrinking trajectory	A	202/6186
444	*GNB1*	chr1:g.1747229	c.169A>G	p.Lys57Glu	exact	4·32	shrinking trajectory	B	244/5654
444	*GNB1*	chr1:g.1747229	c.169A>G	p.Lys57Glu	exact	3·24	shrinking trajectory	C	205/6327
444	*GNB1*	chr1:g.1747229	c.169A>G	p.Lys57Glu	exact	3·27	shrinking trajectory	D	29/887
444	*GNB1*	chr1:g.1747229	c.169A>G	p.Lys57Glu	exact	1·48	shrinking trajectory	E	33/2224
445	*JAK2*	chr9:g.5073770	c.1849G>T	p.Val617Phe	exact	2·47	growing trajectory	A	233/9430
445	*JAK2*	chr9:g.5073770	c.1849G>T	p.Val617Phe	exact	5·16	growing trajectory	B	366/7089
445	*JAK2*	chr9:g.5073770	c.1849G>T	p.Val617Phe	exact	16·94	growing trajectory	C	1572/9278
445	*JAK2*	chr9:g.5073770	c.1849G>T	p.Val617Phe	exact	22·85	growing trajectory	D	745/3260
445	*JAK2*	chr9:g.5073770	c.1849G>T	p.Val617Phe	exact	25·2	growing trajectory	E	530/2103

a) CHDMs in the literature are listed in Table S4a
b) We cannot exclude that some detected somatic mutations with very low VAF are false positives
c) Average variant and total read counts for PCR1 and PCR2. Generated by mpileups of all non-error-corrected reads; insertions and deletions are manually checked for read counts in the IGV browser

Supplemental Figures

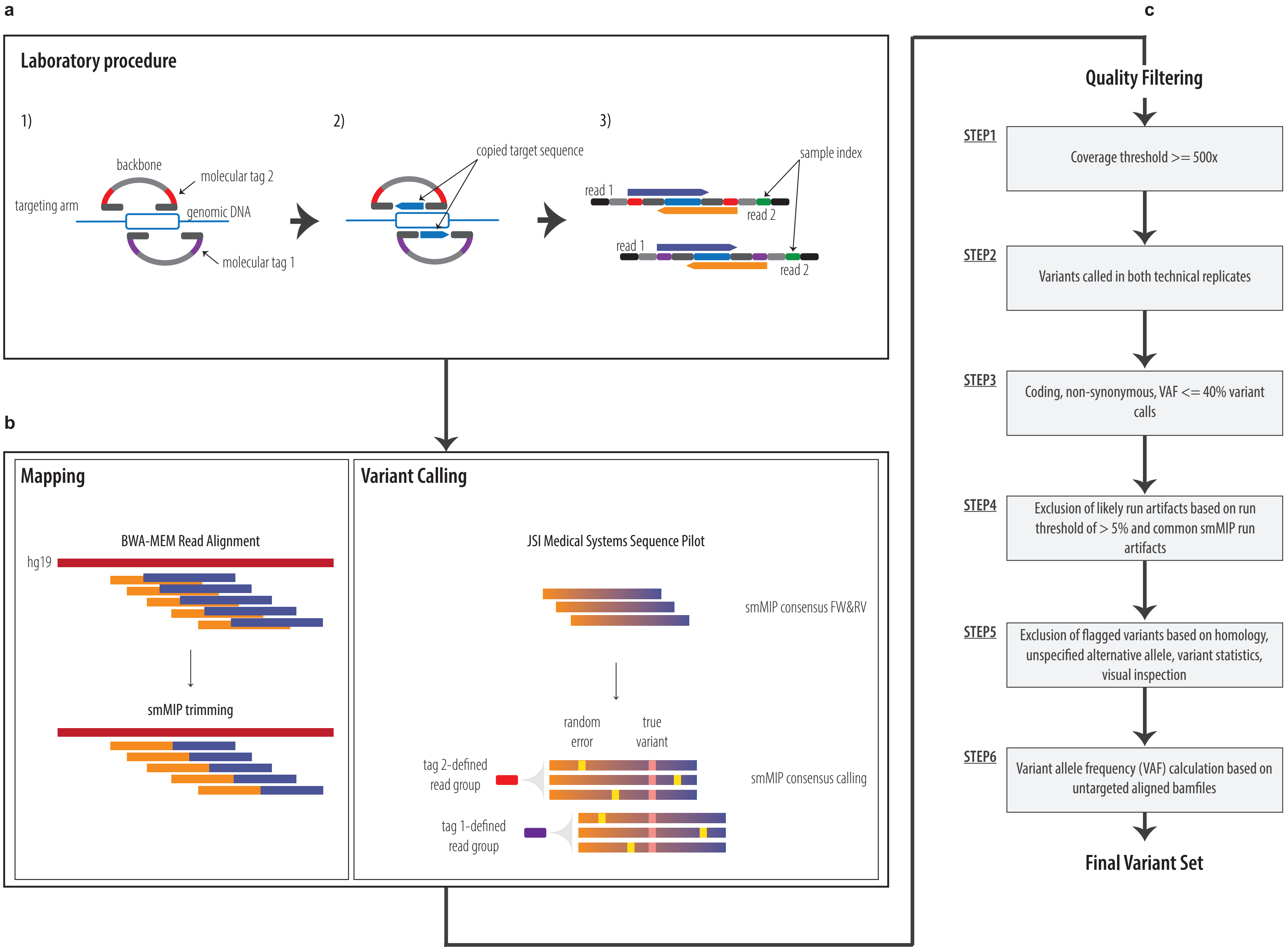

Figure S1: **smMIP workflow. (a)** (adapted from Hiatt et. al. 2013) smMIP insert-sizes were shortened to 54nt to enable full forward and reverse read coverage (double sequencing of each insert), target-sequences were generally targeted by at least two independent smMIP-probes (double tiling), and each smMIP capture underwent two idependently barcoded PCR-reactions (double PCR replicates). Sequecing was performed using the Illumina NextSeq 500 system. **(b)** (adapted from Hiatt et. al. 2013) Raw sequencing data was converted to FastQ-files after which two independent data processing strategies were applied. Mapping: FastQ-file reads were aligned to the reference genome (Hg19) with BWA-MEM after which the overlap between Forward (FW) and Reverse (RV) reads was trimmed off (smMIP trimming). Variant Calling: FastQ-files were imported in JSI Medical Systems Sequence Pilot in which first a consensus between FW and RV reads is determined (smMIP consensus FW&RV), after which tag-defined read groups enable smMIP consensus calling. **(c)** The aligned reads and resulting variant calls are then subjected to a stringent quality filtering pipeline. First, individuals with an average sequencing depth <500x are excluded (STEP1). Second, only variants called in both technical replicates are kept (STEP2). Third, coding, non-synonymous variant calls with a Variant Allele Frequency (VAF) <= 40% are kept (STEP3). Fourth, likely run-specific artifacts (variant calls in > 5% of samples per run) are excluded (STEP4). Fifth, exclusion of variants based on homology, unspecified alternative allele, variant statistics, and visual inspection (STEP5). And finally, for each variant position we generated mpileups based on the aligned reads to determine the final VAF for each CHDM (STEP6).

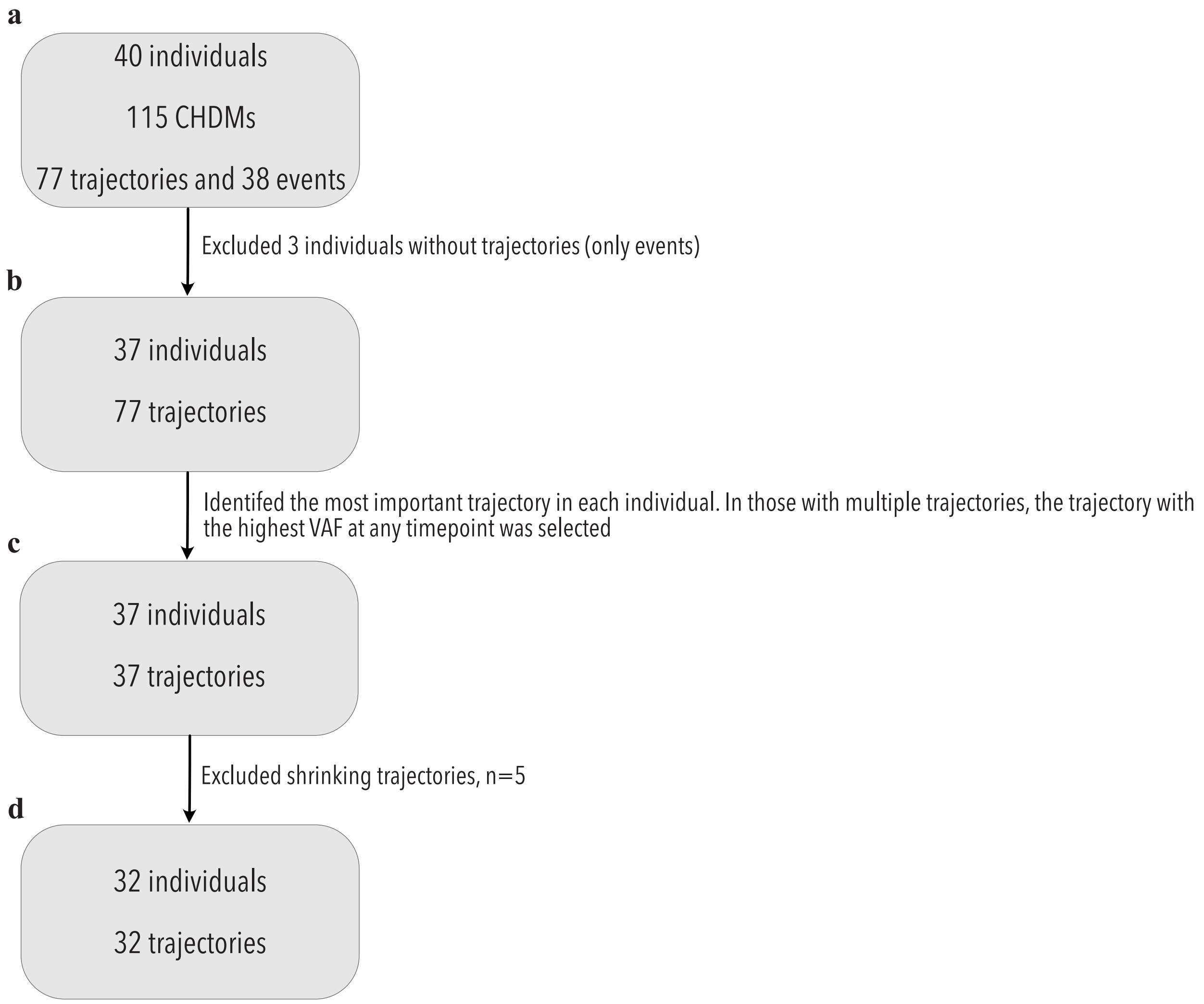

Figure S2: **Trajectory selection flowchart. (a)** In the 40 individuals in our multiple-timepoint obesity dataset, we identified 115 candidate clonal haematopoiesis driver mutations (CHDMs), corresponding to 77 trajectories (i.e., the same mutation identified at 3 or more timepoints) and 38 events (*i.e.,*the same mutation identified at only 1 or 2 timepoints). **(b)** Trajectories were present in 37 individuals, while 3 individuals without trajectories were excluded. **(c)** We next selected the trajectory with the highest variant allele frequency (VAF) at any timepoint in each individual, leaving 37 trajectories in 37 individuals. **(d)** Finally, we excluded individuals in which the selected trajectory was shrinking, leaving 32 trajectories in 32 individuals for analysis.

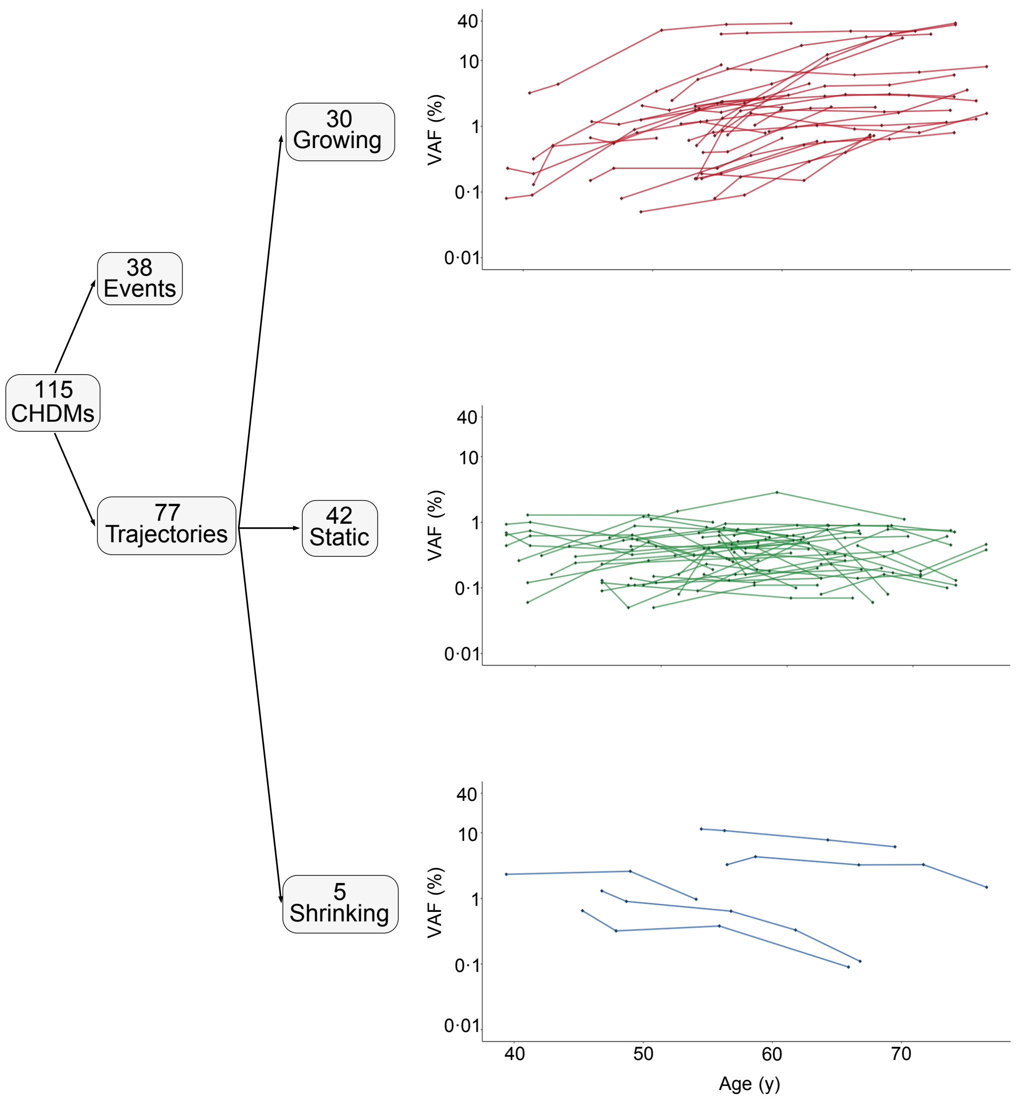

***Figure S3*: Categorisation of CHDMs over 20 years.** Categorisation of CHDMs into events (occurring only once or twice) and trajectories. Trajectories are further categorised into growing, static and shrinking, and their VAFs over time are shown.

Supplemental References

1. Acuna-Hidalgo R, Sengul H, Steehouwer M, van de Vorst M, Vermeulen SH, Kiemeney L, Veltman JA, Gilissen C, Hoischen A. Ultra-sensitive Sequencing Identifies High Prevalence of Clonal Hematopoiesis-Associated Mutations throughout Adult Life. Am J Hum Genet 2017;101:50-64

2. Bray GA, Kim KK, Wilding JPH, World Obesity F. Obesity: a chronic relapsing progressive disease process. A position statement of the World Obesity Federation. Obes Rev 2017;18:715- 723

3. Li H, Durbin R. Fast and accurate short read alignment with Burrows-Wheeler transform. Bioinformatics 2009;25:1754-1760

4. Eijkelenboom A, Kamping EJ, Kastner-van Raaij AW, Hendriks-Cornelissen SJ, Neveling K, Kuiper RP, Hoischen A, Nelen MR, Ligtenberg MJ, Tops BB. Reliable Next-Generation Sequencing of Formalin-Fixed, Paraffin-Embedded Tissue Using Single Molecule Tags. J Mol Diagn 2016;18:851-863

5. Weren RD, Mensenkamp AR, Simons M, Eijkelenboom A, Sie AS, Ouchene H, van Asseldonk M, Gomez-Garcia EB, Blok MJ, de Hullu JA, Nelen MR, Hoischen A, Bulten J, Tops BB, Hoogerbrugge N, Ligtenberg MJ. Novel BRCA1 and BRCA2 Tumor Test as Basis for Treatment Decisions and Referral for Genetic Counselling of Patients with Ovarian Carcinomas. Hum Mutat 2017;38:226-235

6. Li H, Handsaker B, Wysoker A, Fennell T, Ruan J, Homer N, Marth G, Abecasis G, Durbin R, Genome Project Data Processing S. The Sequence Alignment/Map format and SAMtools. Bioinformatics 2009;25:2078-2079

7. Hiatt JB, Pritchard CC, Salipante SJ, O'Roak BJ, Shendure J. Single molecule molecular inversion probes for targeted, high-accuracy detection of low-frequency variation. Genome Res 2013;23:843-854
